# Shifting Perceptions about Microbes and Scientists: Reflections on Activities with High School Students

**DOI:** 10.1093/iob/obag011

**Published:** 2026-03-26

**Authors:** E Funk, A Lasek, K A Schmidtke, A J Reese

**Affiliations:** College of Arts and Sciences, University of Health Sciences & Pharmacy, St. Louis, MO 63110, USA; College of Arts and Sciences, University of Health Sciences & Pharmacy, St. Louis, MO 63110, USA; College of Arts and Sciences, University of Health Sciences & Pharmacy, St. Louis, MO 63110, USA; College of Arts and Sciences, University of Health Sciences & Pharmacy, St. Louis, MO 63110, USA

## Abstract

Engaging students in science influences not only their knowledge but also their interest in becoming part of a scientific community. Where people hold negatively stereotyped views, they are unlikely to appreciate communications of nuanced findings, e.g., that the category of “microbes” is composed of both disease-causing and good/useful agents. This curriculum article describes three activities that could help students consider positive and negative microbial agents and who does science. The first activity aims to help students learn about disease-causing microbes. Here, 25 stuffed microbes were placed around the room with labels. Students were given a handout with a matching activity that they could complete by reviewing the labels on the stuffed microbes. Students were encouraged to explore, learn, and record their answers in small groups. To go over the results, students generated a bingo board and reviewed the answers with the instructor. The second activity focused on good/useful microbes. Here, students watched a video and a slideshow, and discussions about microbes involved in a range of useful and often health-promoting functions. The third activity introduced a diverse range of living scientists. Here, pictures of 12 living scientists were posted in a hallway with stories. Students engaged in a scavenger hunt that prompted them to find key features within these posters and solve a word puzzle. Students filled out assessments before and after the activities, asking about how many disease-causing agents they knew, how many good/useful agents they knew, and how they would describe a scientist. After the activities, students reported being able to name more bad and good/useful microbial agents. Additionally, more students described scientists with positive stereotyping language. Students expressed that they enjoyed the activities. Thus, engaging students in these three activities can shift self-reported knowledge about microbes and perceptions about scientists in a positive direction. Our discussion explores how these activities could translate to other student populations or scientific topics.

## Introduction

While members of the general public are aware of the association between microbes and disease at a broad level, they are less aware of different types of microbes and the positive roles they can play (Kokkinias et al. 2024). Part of this gap likely stems from salient personal experiences with disease-causing microbes, e.g., causing them to miss important events or to lose people they care about (Timmis et al. 2019). Less attention has been given to the positive roles microbes can play. To address this gap, there has been a push towards “positive microbiology” in microbiology education (Oli 2023). School sessions present an opportunity to engage students in longer conversations about both disease-causing and good/useful microbial agents that could shift their perceptions. Many college majors offer coursework in microbiology, and strategies have been devised to promote fun and engaging learning activities (Webb 2015). However, negative views of bacteria can persist, and more work is needed to help students consider the positive roles microbes can play (Lane, Momsen, and Wiltbank-Chau 2025). In the current curriculum article, we focus on shaping positive attitudes towards microbes before students enter college, i.e., high school students, such that they are ready to consider such information from their first semester in college.

As we push towards “positive microbiology” in microbiology education, we also attend to how students view scientists (Dolan et al. 2012). Students’ understanding of who does science influences whether they attend to and learn from scientific communications, and whether they see themselves as scientists in the present and future. Students’ understanding of who does science has been examined by asking them to draw pictures of scientists for over 50 years. Recent meta-analyses of the Draw-A-Scientist Test (also known as DAST) suggest that while younger students (especially girls) are drawing more female scientists than in the past, older students are still drawing more male scientists (Miller et al. 2018). This underscores the continued need to introduce students in high school and college to a range of scientists from a full spectrum of backgrounds (including but not limited to gender diversity) to help them imagine anyone, everyone, and themselves as possible scientists.

A suite of articles nicely lay out the importance and background of why we should be addressing science stereotypes in the 21^st^ century, the history of science stereotypes and their persistence, the value of addressing these at various school settings, and the role teachers and faculty can play in providing counter-stereotype examples (Schinske, Cardenas, and Kaliangara 2015; Schinske et al. 2016; Ovid et al. 2023). Here we will focus on interventions at the college, high school, or outreach level that address how students consider who does science and whether or not fields of science could be for them.

In work looking at student perceptions of scientists, researchers examined students’ written descriptions (Schinske, Cardenas, and Kaliangara 2015). They asked participants of a Human Biology course to respond to the following item: “Based on what you know now, describe types of people that do science. If possible, refer to specific scientists and what they tell you about the types of people that do science.” Students’ responses were coded into two categories, including types of stereotypes (positive, negative, or stereotypical scientist names) and types of non-stereotypes (non-stereotype descriptions or non-stereotypical scientist names). In this population (largely identifying as non-white) they found that positive stereotypes were more common than negative stereotypes (Schinske, Cardenas, and Kaliangara 2015). The most common non-stereotype response expressed identified was that “any type of person” can do science, and students who described scientists in such non-stereotyped tended to earn higher course grades. Thus, student perceptions about “who does science” can influence their academic achievement in biology courses.

In another effort to influence students’ perceptions, researchers developed a semester-long “Scientist Spotlight” activity of readings and reflections that allow students to explore who contributes to science, including voices often not included in textbooks (Schinske et al. 2016). This activity helped college students consider counterstereotypes of scientists and enhanced their personal science identities (Schinske et al. 2016). Other researchers have found that this type of activity can be particularly impactful for first-generation and women students (Metzger, Dingel, and Brown 2023). Where possible, such activities should be combined with strategies that humanize the scientists (Costello et al. 2025), e.g., by showing that scientists share similar values, interests, and experiences as the intended audience. In one study (Schultheis et al. 2024), researchers compared the effects of Scientist Spotlight posters containing only control information (research background), to those containing control information plus a photo, and those containing control information plus photo and additional humanizing information. Students were twice as likely to relate to scientists presented in the third group than the others (Schultheis et al. 2024). Thus, humanizing information about who does science can influence student perception.

All of the aforementioned studies have focused on college students. This left us wondering: what about similar activities for high school students? Some research has been conducted here on this topic. In 2023, Ovid et al. transformed a version of the “Scientist Spotlight” into a semester-long study with high school students. Positively, they observed that high school students who participated could better relate to scientists and were more likely to express seeing themselves as scientists (Ovid et al. 2023). This is promising, but implementation of a semester long activity is a hard sell for many high school teachers, who undoubtably have a lot of information to cover already. Briefer interventions may prove easier to implement. For example, in a virtual DNA-day university outreach activity, researchers found a positive shift in high school student perceptions about science (Barry et al. 2023). This is the type of brief activity that we are describing in this current curriculum piece.

With this article, we describe three curriculum activities designed to be accessible, transferable, and safe for any high school student. Activity 1 involved learning about disease causing microbes, Activity 2 involved learning about the positive role microbial agents can play, and Activity 3 was a modified version of a Scientist Spotlight project. We wanted the three activities to help students be curious about science, to encourage them to be excited about learning, and to have them walk away thinking about who does science differently than they did when they began the set of activities.

Accompanying our set of three activities are three research questions: 1) Can engagement in Activity 1 increase the number of disease-causing microbial agents that students can name? 2) Can engagement in Activity 2 increase the number of good/useful roles for microbial agents that students can name? and 3) Can engagement in Activity 3 shift the student perceptions of who does science? These questions lie in the intersections of the discipline of microbiology and the realm of biology education, as they explore ways to engage and teach students content, while supporting student identity within science fields. We offer them up to add to the collection of Discipline-Based Education Research (DBER) strategies that “develop evidence-based knowledge and practices that improve teaching and learning in the science, technology, engineering, and mathematics (STEM) disciplines.” (Henderson et al. 2017) and, in particular, to help students think broadly of who participates in the science enterprise and see themselves as being able to contribute.

## Methods

A pre-post evaluation was conducted with students completing worksheets before and after all three activities. The activities were conducted as part of a summer immersion program that enrolled high school students for a week-long campus experience. Most students were rising juniors or rising seniors and were from around the United States, particularly from Illinois, Missouri, Texas, and Oklahoma. The activities were delivered for two consecutive summers on a Missouri campus. In 2024, 23 students took part in a 2-hour session. In 2025, 27 students took part in a 2.5-hour session; the extra 30 minutes were added to encourage greater engagement in our Scientist Spotlight poster activity.

All students worked together to complete the activity with disease-causing microbes (activity 1). Then, students were split into two smaller groups. While one group watched a video about body microbes (part of activity 2) and then viewed posters (activity 3), the other group engaged in the reverse order. Finally, all groups came together for a concluding slideshow and student discussion about the roles of good/useful microbes (the remaining part of activity 2). The session ended with a concluding discussion summarizing what students had learned. At the conclusion of both summer immersion programs, students were invited to write notes of appreciation to participating faculty. Each activity is described below with the corresponding research question.

### Research question 1) can engagement in activity 1 increase the number of disease-causing microbial agents that students can name?

**Fig. 1:**
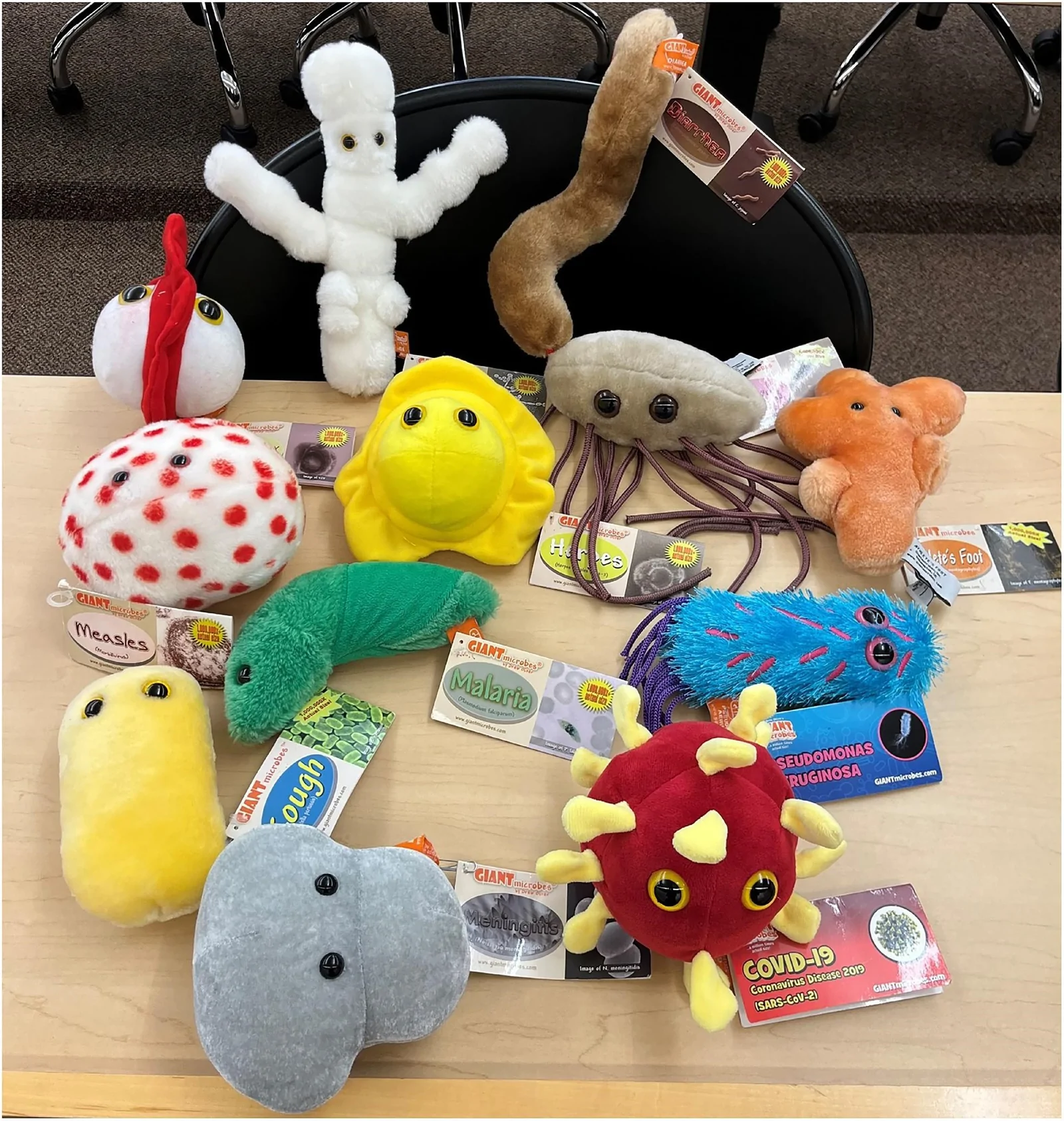
Stuffed microbes (available from giantmicrobes.com and other distributors that sell their stuffed products) with information tags, such as these shown here, were set up around the room for students to read and explore. A paper matching activity included descriptions of microbes and a word bank of their names for students to then select for matching. Nuances of the different types of microbial agents (e.g. bacteria vs. viruses) can be discussed during an activity review.

The first activity aimed to help students identify types of microbial diseases and the different types of microbial agents that cause them. We recognize that often “germs” are all conceptually lumped together, and we wanted to lay a foundation to help students see they aren’t all the same type of agent and to begin to disentangle the different groups of microbial agents (such as bacteria, viruses, fungi, protozoa, etc.). There were 25 “fuzzy microbes,” i.e., stuffed animals (giantmicrobes.com) with name and information labels stationed around the room (Fig. 1). A paper matching activity with descriptions of all 25 microbes was printed on one side of a handout, and the flip side had a bingo board grid with a microbial-name word bank of all the microbes used in the activities (see supplemental material I). Students were encouraged to move around the room in small groups to explore, learn, and record the different microbial-name answers matching the disease agent descriptions. Then, students used the list of microbes to create their own bingo board, and we reviewed the answers by playing bingo. For this, the instructor read the description from the matching activity, and students could report microbial names (or common names) or hold up the appropriate stuffed microbe near them to respond. In this way, students could give answers in an engaging way. During this portion of the activity, the instructor also discussed differences in the types of microbial agents. Microbial stickers were provided for bingo participation prizes.

### Research question 2) can engagement in activity 2 increase the number of good/useful roles for microbial agents that students can name?

The second activity aimed to facilitate students’ ability to recognize that not all microbes are disease-causing and to consider some good/useful roles microbes play. The value of microbiomes and microbial balance in the human body was introduced with a five-minute NPR video “The Invisible Universe of the Human Microbiome.” (https://www.youtube.com/watch?v=5DTrENdWvvM). In the debriefing, students were asked to share something they learned from or were surprised about from the video. The content of the video was later paired with a slideshow (see supplemental materials II) about microbes involved in a range of good/useful activities. The slideshow was inspired using images from a coloring book produced by the American Society for Microbiology in 2024 that portrayed topics (see Table 1), similar images could be produced using online tools like Canva or stock images. Briefly, the images covered microbes all over and their role in the origin of life; their use in food, agriculture, household products, research, and medicine; and even the beauty/art with them (for more details, see Table 1). In 2024, copies of the coloring book were provided to participants, but we were unable to obtain the books again to pass out in 2025. The choice of the coloring book images was to make them accessible and engaging for students. In addition to any text accompanying the coloring book images, many slides contained question prompts to engage the students in dialogue with the instructor, such as “As you saw in the video, microbial cells outnumber our body cells about 10 to 1. Have you heard of that before? Does it make you consider what makes you YOU?” or “Do you know of other foods that make use of microbes in some way?” In addition to showing images, slides prompted instructor-led discussion with students about various adaptations microbes may need to function in the extreme conditions of some locations, or products that they make that humans have developed for medicines or making our lives easier. The slideshow also featured additional fuzzy microbes or microbial product props to help students connect microbiology and microbial products they could see in everyday life. This type of discussion invited students to contribute what they knew or were curious about regarding these topics.

**Table 1:** **Images of microbial roles used in these activities were grouped by topic areas. ** These examples were inspired by the coloring book by American Society for Microbiology.

Topics Covered	Type of Image Examples
Microbes & our bodies	Bodies, *E. coli* vitamin production in gut
Role in food production	Yogurt, cheese, and bread
Role in agriculture	Enhance crop growth and protect crops from diseases
Microbes can be everywhere, role in bioremediation	Water, land, air, oceans, maybe even space
Role in production of enzymes, such as from *Thermus aquaticus* used in labs	Volcano
Role in household products	Laundry detergent
Life origins and model organisms	Early planet life, lab studies to demonstrate learning from them
Source of medicine	Antibiotics, insulin production, and vaccines
Source of beauty and art	Different colors, shapes, and beauty in agar art creations

### Research question 3) can engagement in activity 3 shift students perceptions of who does science?

The third activity aimed to help students consider science in a fun context that anyone could be part of now and in the future. While this activity could have been focused exclusively on featuring microbiologists, it was purposely broader to help students feel like there was a place for them in science even if they weren’t interested in tiny microbes. We thought this might help add a bit of “something for everyone” to the outreach event. It gave us the opportunity to blur the lines between microbiology and other fields of science. Expanding to a larger collection and representation of “who does science” also allowed us to make use of a collection of posters that had been created for a previous event (Hunt 2023).

For this activity, we used 12 16 × 20-inch posters presenting different living scientists from different fields (see Table 2 for the types of scientists featured; Reese 2023 unpublished; Hunt 2023). Featured scientists were each contacted to approve the text and supply images for use in the posters. The goal of the posters was to humanize scientists as accessible people. To do so, each poster included a photo of a different scientist, along with their name and job title, and a brief plain-English summary describing their job, background, and how they got into science. For participants who wanted to learn more about a particular scientist, a QR code was also available to scan (see supplemental material III for a poster layout example, used with permission of the featured scientist). Living scientists were purposely selected to help provide a counterstereotype to the idea of scientists being of the past. Students were invited to see aspects of themselves in the scientists as they discovered shared interests, struggles, or successes. To encourage engagement with the posters, a scavenger hunt paper activity (see supplemental material IV) took place, inspired by ones associated with the If/THEN #IfThenSheCan exhibit of women scientists’ 3D statues (https://ifthenexhibit.org/exhibit_preview/). In 2024, students were not provided with the scavenger hunt paper guide. In 2025, students were given this guide to help students purposely connect with the material contained on the posters.

**Table 2:** **Scientists featured in posters addressed a range of science fields and backgrounds. ** Living scientists were featured with current scientific topics and scientists. Aspects of each scientist’s background and work were shared to help students connect with them in various ways.

Scientist field	Scientist background, identity, interest highlights
Pediatrician	Black woman who co-founded “We are DocMcStuffins” and appeared on the show
Otolaryngologist	Man of Asian ancestry, musician, connected musical interested with treating hearing issues
Cosmologist	Seasoned white woman who has used the Hubble telescope to help us understand the universe
Chemistry professor	Black gay man who has used TikTok to help public reimagine chemistry and science
RNA biochemist	Hungarian woman who persevered in science, despite loss of jobs and funding, to understand the mechanisms of RNA
Molecular biologist	Mexican-American woman whose work led to insulin production
Citizen science	White woman and former NFL cheerleader who recruited other cheerleaders to engage public in science
High School genetics teacher	Trans-man who helps scientists with genetics curriculum to move beyond binary constructs
Pharmacologist	British woman with a learning disability who is helping STEM be more neuroinclusive
Microbiologist	Spanish woman who is leading work in how bacteria can clean up the environment
Science entrepreneur	Muslim woman using biotechnology to make accessible diagnostic tools
Zoologist	White woman protecting turtles and involved in conservation biology

### Worksheets and feedback

Pre-post evaluations were collected via paper worksheets, in person, before and after all three activities took place. Student responses were linked to allow for student-level pre-post evaluation. The first worksheet question asked students to report the number of disease-causing agents they could name. The second worksheet question asked students to report the number of good/useful roles for microbes they could name. The last worksheet question asked students to describe what a scientist looks like. During the first year, students only provided written responses to the third question. During the second year, one student asked if they could draw a picture. The instructor said yes, and other students took up the opportunity to draw as well.

Overall, feedback about the sessions was gathered from student notes of appreciation. The opportunity to provide a note was offered by the summer immersion team at the end of all the sessions students attended. Only information relating to our activity was provided to the research team. This information is not linked to their pre-post evaluation worksheets.

### Evaluation

To see if the self-reported numbers of “bad microbes” or “roles for good/useful microbes” increased after the activities, we compare the numbers of students reporting more than two before and after the evaluation. Then, Wilcoxson’s sign-ranked test is used to assess whether these self-reports increased significantly.

To see if students' perceptions of scientists changed, two researchers (EM and AL) reviewed students’ written responses/drawings, and coded each according to its affective value (either negative, neutral, or positive) and who it represented as doing science (either “science is not for everybody” or “science is for everybody”). This strategy is informed by approaches used in previously published studies (Schinske et al. 2016; Metzger, Dingel, and Brown 2023) and inductively revised to better reflect our data. Then, they met to discuss their decisions with the instructor, and disagreements were settled through consensus discussions. Bar charts displaying the proportions of responses for each agreed code are provided to assess changes from pre to post visually. Exact McNemar’s tests are used to assess whether the proportion of students expressing neutral or positive stereotypes increased after the activities, and whether the proportion of students expressing that science is for everybody increased after the activities. All statistics were conducted using SPSS version 28.

Overall feedback is described, with examples provided.

## Results

### Research question 1) can engagement in activity 1 increase the number of disease-causing microbial agents that students can name?

Descriptively, students reported being able to name more disease-causing microbes after the activity. Before the activity, 24/50 students (48%) said they could name more than two disease-causing microbes. After the activity, 49/50 students (98%) did. The Wilcoxon test indicated that their perceived knowledge significantly increased (Z = 6.07, p < 0.001).

### Research question 2) can engagement in activity 2 increase the number of good/useful roles for microbial agents that students can name?

Descriptively, students reported being able to name more good/useful roles for microbes after the activity. Before the activity, 6/50 students (12%) said they could name more than two useful/good roles for microbes. After the activity, 45/50 students (90%) did. The Wilcoxon test indicated that their perceived knowledge significantly increased (Z = 6.04, p < 0.001).

### Research question 3) can engagement in activity 3 shift the student perceptions of who does science?

Student responses to the third question, asking what a scientist looks like, were brief, typically less than 10 words long. Responses coded as characterizing scientists negatively included phrases like “crazy hair.” Examples of neutral phrases included “Someone who studies science.” Examples that were coded as positive stereotypes included “A person who is doing their best to better the future world.”

**Fig. 2:**
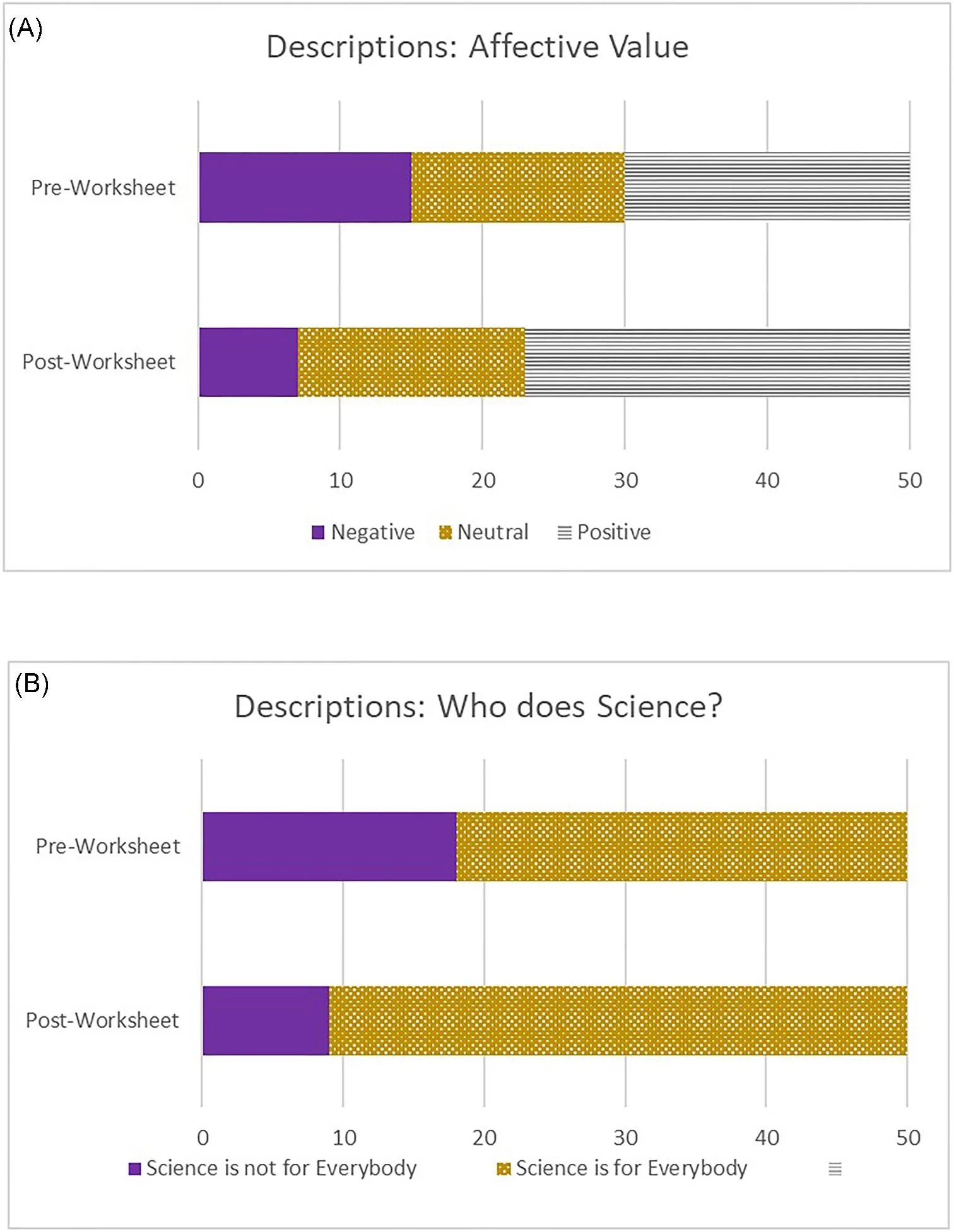
More students described scientists with more positive stereotyping descriptions and indicated that science could be done by normal people or everyone after the activities. Examples of descriptions and how they were coded are provided in the text. Images include data from both 2024 and 2025 surveys. (A) Affective Value graph demonstrates how student descriptions of scientists were coded. (B) Who Does Science? graph shows how student descriptions were coded surrounding how they perceived who are scientists.

Chart A in Fig. 2 displays the proportion of students coded as characterizing scientists by each affective value. The proportion of negative descriptions decreased, and positive descriptions increased. Eight (of the 50) students changed from expressing negative stereotypes to neutral or positive stereotypes. No changes occurred in the other direction. The exact McNemar’s test determined that the proportion of students expressing neutral or positive stereotypes increased after the activities, p < 0.01.

The same responses were coded regarding who does science. Examples of phrases coded as science is not for everybody include “Bill Nye the Science Guy” and “stethoscope, white coat, name tag.” Examples of phrases coded as science is for everybody include “someone interested in researching” and “people from all over the world with different backgrounds.”

Chart B in Fig. 2 displays the student responses characterizing who does science. Here we see more students shifting to see science as open to everyone after the activity. Nine (of the 50) students changed from expressing that science was not for everybody to everybody. Again, no changes occurred in the other direction. The exact McNemar’s test indicated a significant increase, p < 0.01.

### Results—overall feedback

In their voluntary thank you notes for the activities, students expressed that the activities were interactive and fun, that they were anxious initially about a multi-hour block but had a good time, and that they were excited to learn more microbiology. Example phrases from this feedback included the following: “Thank you for taking time out to give us a lecture about microbiology. I didn’t really know about it” and “I loved seeing all the posters of scientists and reading about their lives.”

## Conclusions and further discussions

The observed shifts in perceived knowledge about microbes and perceptions about who does science suggest that even a few hours can influence how students relate to this material, at least in the short-term. This underscores the potential value of classroom or outreach activities of this nature and on these topics. In conclusion, a brief set of activities can be used to impact high school students' perceived awareness about microbes and perceptions of who can do science. This, in turn, could lead to them being more open to considering broader future understandings of the variety of microbes and their impact, as well as a range of science fields and scientist stories as they grow and learn.

The initial two activities are born out of a more traditional information-delivery model of science education in which a scientist’s job is to provide accurate information that their audience can use as citizens and engaged members of society (Oreskes 2022). For instance, if people know more about good and useful microbes, they might be more open to receiving new medications and products that are drawn from them. They may be more aware of the value of good microbes and their roles in training our immune systems and in our gut health. If people understand more about bad microbes and how they are spread, they may be better able to value methods of keeping themselves and others healthy, such as handwashing, avoiding others when they are ill, and the value and importance of vaccines.

Reflecting on our set of three activities, we note that the third activity does something different by engaging the participant with information about the scientist and allowing them to see them as people. A more nuanced appreciation of decision-making recognizes other deliberate and non-deliberate factors that play a role in what people ultimately do (Kahneman, Lovallo, and Sibony 2011; Dolan et al. 2012). For instance, the messenger effect describes how the perceived credibility, authority, and relatability of the person delivering information can be more influential than the content of the message itself. Since “othering” scientists can reinforce beliefs that scientists are biased in ways the audience perceives to be unfavorable (Beauchamp, Roberts, and Piper 2022), this type of approach to humanize scientists can make them more relatable. Shifting students’ perceptions about who does science, in a way that could include people with whom they feel they can connect in some way, could help them more fully consider the messages scientists share and be more interested in contributing to fields of science themselves.

### Limitations

Limitations to the work include that the numbers of familiar microbes were self-identified, and participants were not requested to verify their responses by listing examples. Social desirability bias may have influenced participants such that they did not feel comfortable reporting lower numbers or that their belief about who does science did not change. Different prompts or instructions may have told us more about what students learned and who they believe does science. We also do not have any further follow-up with students about their recollection of microbe numbers or current descriptions of scientists. It is possible that the short-term benefits found in the current evaluation would not endure after students' experience at the summer immersion program ended.

The group sizes were between 20 and 30, and the students voluntarily committed to participating in week-long college camp at a science institution. This small convenient sample of students may have been more receptive to our activities; it is uncertain if these results will generalize to students that are mandated to attend. As no demographic information was collected, we cannot comment on whether different demographic groups are more or less likely to benefit from these activities.

### Applications for other settings

Variations on these activities could be employed to fit different audiences and delivery contexts. Our slide deck is provided as supplemental materials, and we invite users to modify it to fit their audience and delivery context. Instructors with already tight schedules could choose to integrate just one or two of the activities. Alternatively, instructors building a new curriculum could expand the activities into three separate days with greater opportunities for personal reflection or assessments that integrate information from the lesson more directly. For example, our matching task about disease-causing microbes was an open-ended and collaborative project to introduce students to these organisms, similar matching tasks could be used as review sessions (open or closed notes) for students to practice their understanding or to test their understanding of key microbes in medicine. While we did not include a more objective assessment of student learning, these could be created. Such assessments should be informed by their intended audience, likely requiring deeper demonstrations of understanding from college students (e.g., reflective writing/presenting) and a greater focus on recall from high school students (i.e., multiple-choice/free-recall tests).

In our methods, we described students' desire or impulse to draw pictures rather than write descriptions of scientists. Where possible, such drawings could be used to facilitate conversations about the material presented during which students can hear and share their diverse perspectives. Pictures could also be provided, and students (particularly younger ones) could be prompted to “pick everyone who looks like a scientist” from a set. While this could be done in various ways, all images could in fact be of scientists. On the other hand, prompts that elicit more specific verbal descriptions would allow students to articulate what they mean and not require interpretation when coding.

Our activities were conducted in a university building where we regularly work, where acquiring stuffed microbes and creating Scientists Spotlight posters is possible, as is finding a space to store and display them. Where this is not possible, printed or virtual images could be used to replace or supplement these physical displays at, e.g., festivals, museum nights, virtual fieldtrips, etc., where they may reach a broader audience. While our Scientist Spotlight posters purposedly focused on a wide range of science disciplines, scientists could be selected to more narrowly highlight certain fields of science or a range of scientist backgrounds, personal identities, and interests to the intended audience.

### Resources for other settings

Several resources exist that could be used to feature a range of scientists, including sites like the Scientists Spotlight Initiative (https://scientistspotlights.org/), American Society for Microbiology (ASM) spotlights (https://asm.org/browse-by-keyword/spotlight) or the Howard Hughs Scientist Spotlights videos collection project of scientists (https://www.youtube.com/playlist?list=PLqwpOkZ9dxzKvyeKxd1RtEwyKJTJxNsMN).

There are a number of books that highlight multiple female scientists who could be featured (Ignotofsky 2017; Whitaker and Barton 2018; Shlian 2023). For a younger audience, the site A Mighty Girl (http://amightygirl.com) provides a “collection of books, toys and movies for smart, confident, and courageous girls” and boys that could be used to support learning about scientists. Table 3 provides a starter list of resources for finding scientists to highlight from specific identities such as Black or African American, Hispanic or Latinx, Indigenous, Native American or First Nation, disabled or persons with disabilities, or LGBTQIA + in STEM. These are not meant to be exhaustive lists but could be valuable to find individuals studying science topics that may be of interest to different student groups with different perspectives. Additional resources support comprehensive approaches to populations within science (Johnson and Okoro, 2016; Cooper, et al. 2020; Powell, Terry, and Chen 2020; Peterson, 2021; Cox, Elliot, and Harris, 2021; Vasquez, 2024; and websites such as http://sacnas.org and http://aises.org).

**Table 3:** **Selected resources for highlighting scientists with different identities.** These resources could be used to generate collections of scientists for different groups of students to consider and with whom they may relate. The list is not intended to be exhaustive, but rather to provide three starter locations for someone new to scientists who identify in these ways.

Scientist identity	Selected resources for scientists
**Black & African American**	1. New York Public Library list of Black History STEAM books https://www.nypl.org/blog/2021/02/01/black-history-steam-booklist 2. Gladstone Institutes slide show and timeline https://gladstone.org/index.php/news/highlighting-black-scientists-past-present 3. Science Buddies description of 40 Black Scientists https://www.sciencebuddies.org/blog/black-history-month-scientists
**Hispanic & Latinx**	1. Cell Press list of 100 hispanic/latinx scientists and engineers https://crosstalk.cell.com/blog/100-inspiring-hispanic-latinx-scientists-in-america 2. Gladstone Institutes spotlights latinx scientists https://gladstone.org/news/spotlighting-latinx-scientists 3. Science Buddies focus on hispanic scientists https://www.sciencebuddies.org/blog/hispanic-scientists-engineers
**Indigenous, Native American & First Nation**	1. Advancing Indigenous Science and Engineering (AISES) resource for and about Indigenous science https://aises.org/ 2. Gladstone Institutes highlights Native American scientists https://gladstone.org/news/highlighting-native-american-scientists 3. Science Buddies covers Native American Scientists https://www.sciencebuddies.org/blog/native-american-scientists-engineers
**Disabled, persons with disabilities**	1. Inclusivity for All site https://disabledinstem.wordpress.com/ 2. Celebrating scientists with disabilities https://royalsociety.org/about-us/who-we-are/diversity-inclusion/case-studies/scientists-with-disabilities/ 3. 13 disabled scientists you should explore and discover https://disabilityhorizons.com/2024/05/13-disabled-scientists-you-should-explore-and-discover/
**LGBTQIA+**	1. 500 Queer Scientists https://500queerscientists.com/ 2. Out of the Closet, into the Lab: Five LGBTQ Scientists https://www.mskcc.org/news/out-closet-lab-five-lgbtq-scientists-share-their-stories 3. LGBTQ+ people in STEM https://www.liverpoolmuseums.org.uk/stories/lgbtq-people-stem

Where possible, we encourage instructors/facilitators/teachers to share their experiences and inform us of the ways through which we could positively shift students perceptions of scientists. Microbiology, and indeed all fields of science, need to consider the voices of individuals with all kinds of backgrounds and interests to address present and future challenges. Helping students see how they can be a part of science now and in the future can lead more to explore different fields of science.

## Supplementary Material

obag011_Supplemental_Files

## Data Availability

Student coding and data analysis is available upon request.
