# Supplementary material for: Shifting Perceptions about Microbes and Scientists: Reflections on Activities with High School Students
Source: Integr Org Biol. 2026 Mar 26;8(1):obag011. doi: 10.1093/iob/obag011 (PMC13048273; doi:10.1093/iob/obag011)
Supplement: obag011_Supplemental_Files [file obag011_supplemental_files.zip › Outreach activity instructions, key, microbial activities, & surveys.pdf]

Introduction to Microbiology & Scientists

Instructor resources including

Pg 1 overview of activities
Pg 2 coverage of coloring book and link to NPR body microbe video
Pg 3 matching microbial activity - key
Pg 4-5 matching microbial activity and bingo board that can be printed double-sided for participants
Pg 6-7 pre and post survey (last two pages) that can be printed double-sided and cut in half.

Description: Use of fuzzy microbes from giantmicrobes.com and ASM coloring book to explore some key examples in microbiology (both good and bad), learn something about these agents, explore patterns in their shapes, and make the introduction of microbiology fun and engaging. Scavenger hunt for "bad microbe" information, bingo game for review, etc. would all be strategies to discuss. Range of scientists introduced with posters.

Objectives

1) Identify some of the types of microbial diseases and the agents that cause them.
2) Recognize that not all microbes are bad and consider useful examples.
3) View microbiology in a fun context and that anyone can be a scientist.

Activities:

1) Pre survey
2) Fuzzy microbe round-the-room information scavenger hunt to learn about the different disease-causing microbes and fill in matching.
3) Generate bingo board with microbe name. Go over answers to matching via.
4) Divide into two groups to:
    a. Travel to posters of scientists
    b. NPR video about microbes all around us going on front of room (and scientist photo frame station, optional)
5) Slide show (and coloring books?) to introduce good microbes, with hands-on and additional images to prompt discussion and bring information to daily life context.
6) Post survey

Diverse and good roles in the coloring book:

1) Microbes are everywhere. Around us and inside us. -
2) Microbes are in your yogurt. – Lactobacillus
3) Microbes help us make cheese. – lactic acid bacteria that convert sugars to acid flavors
4) If you like bread, say thanks to microbes from making it. Saccharomyces
5) You need microbes to digest your food. – E. coli
6) Microbes can help crops grow and protect them from diseases.
7) The laundry detergent that cleans your clothes contains products from microbes. – laundry bottle
8) Microbes are under water, on land, in the air, maybe even in space? – bioremediation usage
9) Microbes can live in volcanoes. – Thermus aquaticus role
10) And on the bottom of the ocean. – have to withstand super high water pressure , also, diatoms are marine algae, diatomaceous earth powder comes from fossilized diatoms, which can in turn be used to filter water
11) Microbes started life on our planet. – and may help us with energy in the future with biofuels
12) Some people even think they came from another planet - Martian bacteria
13) Scientists use microbes in the lab
14) We can use microbes to make things like insulin to treat diabetes – insulin package
15) Microbes helped scientists discover the antibiotics you take when you get sick. – Penicillin
16) We can also use microbes to study how diseases, like COVID-19 spread. – covid and covid vaccine
17) Some people even use microbes to make art. – agar art competition link
18) Microbes can make all different types of colors. – metabolism image
19) They can grow in different patterns and shapes – student plate image
20) For all of these reasons, we think microbes are beautiful.

5 min NPR overview of body microbes
https://www.youtube.com/watch?v=5DTrENdWvvM

K.	*Bordetella pertussis* (bacteria)	A. Vaccines help prevent against this cause of meningitis (severe headache and stiff neck with high mortality rate).
U	*Borrelia burgdorferi* (bacteria)	B. This virus causes lesions that "creep" across the skin, if symptoms occur, and outbreaks may continue over time.
P	*Campylobacter jejuni* (bacteria)	C. As the most common cause of fungal skin infections, including athlete's foot, this mold likes warm skin folds.
R.	*Candida albicans* (fungus)	D. Saliva transmission, causes mononucleosis = sore throat, swollen glands, fatigue, and enlarged spleen in young adults.
I	*Chlamydia trachomatis* (bacteria)	E. This agent is found all over & is usually fine but can be drug resistant. Hot tubs or pools not fully cleaned are skin sources.
N	*Clostridioides difficile* (bacteria)	F. A childhood vaccine now prevents this former respiratory-acquired itchy skin infection (causing complications in some).
J	Flu (orthomyxovirus)	G. This agent is the biggest cause of ear infections in children, and is the biggest cause of bacterial pneumonia in adults.
B	Herpes simplex 2	H. This is one of the top causes of food poisoning and is associated with the handling of eggs, poultry, & reptile pets.
S	Human papilloma virus	I. A common sexually transmitted agent, it can damage female reproductive tracts. Causes eye infections globally.
O	Measles (morbillivirus)	J. Respiratory infections that can be severe. Yearly vaccines are developed to maintain immunity (virus changes surface).
D	Epstein-Barr virus	K. Causes whooping cough, a particularly serious cough in infants. Protection from the DTaP vaccine (needs boosters).
X	*Neisseria gonorrheoeae* (bacteria)	L. We've probably each had "strep throat" caused by this agent, though it only causes about 15% of sore throats.
A	*Neisseria meningitidis* (bacteria)	M. Syphilis is a very old disease, but not gone, and on the rise again. Transmitted by unprotected sex, the first stage is sores.
Q	*Plasmodium falciparum* (protozoa)	N. Serious diarrheal infections are associated with overgrowth of this agent after other bacteria are killed by antibiotics.
E	*Pseudomonas aeruginosa* (bacteria)	O. This super contagious virus was life-threatening before a vaccine in 1963. Outbreaks happen where low vaccine rates.
W	Rhinovirus	P. This agent of diarrhea is associated with undercooked food (and is in 80% of poultry) that can severely dehydrate people.
H.	*Salmonella typhimurium* (bacteria)	Q. Transmitted by mosquito types, this agent of malaria lyses red blood cells causing cycles of fevers and chills globally.
V	SARS-CoV-2	R. Most of the time this fungus is no issue, but it can cause skin rashes or genital infections. Resistant strain is an issue.
T	*Staphylococcus aureus* (bacteria)	S. Over 100 types of this virus cause warts, but a few cause cervical cancer. A vaccine has dramatically dropped rates.
G	*Streptococcus pneumonia* (bacteria)	T. "Staph" means grapes which is how these cells cluster. On our skin is fine but in a wound they can cause infections.
L	*Streptococcus pyogenes* (bacteria)	U. Acquired via tick bites, this spirochete causes Lyme disease that may have a bull's-eye rash then aches, fever & fatigue.
M	*Treponema pallidum* (bacteria)	V. Coronaviruses were around before this one, but this one hit the news. Corona refers to the "crown" of protein spikes.
C	*Trichophyton mentagrophytes* (fungus)	W. There is no vaccine to the common cold because there are so many variations. Wash hands to keep these viruses away.
F	Varicella-Zoster virus	X. One of the most common sexually transmitted agents that may not cause symptoms, treated with antibiotics if detected.

	Bordetella pertussis (bacteria)	A. Vaccines help prevent against this cause of meningitis (severe headache and stiff neck with high mortality rate).
	Borrelia burgdorferi (bacteria)	B. This virus causes lesions that “creep” across the skin, if symptoms occur, and outbreaks may continue over time.
	Campylobacter jejuni (bacteria)	C. As the most common cause of fungal skin infections, including athlete’s foot, this mold likes warm skin folds.
	Candida albicans (fungus)	D. Saliva transmission, causes mononucleosis = sore throat, swollen glands, fatigue, and enlarged spleen in young adults.
	Chlamydia trachomatis (bacteria)	E. This agent is found all over & is usually fine but can be drug resistant. Hot tubs or pools not fully cleaned are skin sources.
	Clostridioides difficile (bacteria)	F. A childhood vaccine now prevents this former respiratory-acquired itchy skin infection (causing complications in some).
	Flu (orthomyxovirus)	G. This agent is the biggest cause of ear infections in children, and is the biggest cause of bacterial pneumonia in adults.
	Herpes simplex 2	H. This is one of the top causes of food poisoning and is associated with the handling of eggs, poultry, & reptile pets.
	Human papilloma virus	I. A common sexually transmitted agent, it can damage female reproductive tracts. Causes eye infections globally.
	Measles (morbillivirus)	J. Respiratory infections that can be severe. Yearly vaccines are developed to maintain immunity (virus changes surface).
	Epstein-Barr virus	K. Causes whooping cough, a particularly serious cough in infants. Protection from the DTaP vaccine (needs boosters).
	Neisseria gonorrheoeae (bacteria)	L. We’ve probably each had “strep throat” caused by this agent, though it only causes about 15% of sore throats.
	Neisseria meningitidis (bacteria)	M. Syphilis is a very old disease, but not gone, and on the rise again. Transmitted by unprotected sex, the first stage is sores.
	Plasmodium falciparum (protozoa)	N. Serious diarrheal infections are associated with overgrowth of this agent after other bacteria are killed by antibiotics.
	Pseudomonas aeruginosa (bacteria)	O. This super contagious virus was life-threatening before a vaccine in 1963. Outbreaks happen where low vaccine rates.
	Rhinovirus	P. This agent of diarrhea is associated with undercooked food (and is in 80% of poultry) that can severely dehydrate people.
	Salmonella typhimurium (bacteria)	Q. Transmitted by mosquito types, this agent of malaria lyses red blood cells causing cycles of fevers and chills globally.
	SARS-CoV-2	R. Most of the time this fungus is no issue, but it can cause skin rashes or genital infections. Resistant strain is an issue.
	Staphylococcus aureus (bacteria)	S. Over 100 types of this virus cause warts, but a few cause cervical cancer. A vaccine has dramatically dropped rates.
	Streptococcus pneumonia (bacteria)	T. “Staph” means grapes which is how these cells cluster. On our skin is fine but in a wound they can cause infections.
	Streptococcus pyogenes (bacteria)	U. Acquired via tick bites, this spirochete causes Lyme disease that may have a bull’s-eye rash then aches, fever & fatigue.
	Treponema pallidum (bacteria)	V. Coronaviruses were around before this one, but this one hit the news. Corona refers to the “crown” of protein spikes.
	Trichophyton mentagrophytes (fungus)	W. There is no vaccine to the common cold because there are so many variations. Wash hands to keep these viruses away.
	Varicella-Zoster virus	X. One of the most common sexually transmitted agents that may not cause symptoms, treated with antibiotics if detected.

Student name ____

BINGO REVIEW TIME

Put each of the microbe names in a box below (you can shorten them) and then mark them when you hear their description being read from the matching page.

Bordetella pertussis
Borrelia burgdorferi
Campylobacter jejuni
Candida albicans
Chlamydia trachomatis
Clostridioides difficile
Flu (orthomyxovirus)
Herpes simplex 2

Human papilloma virus
Epstein-Barr virus
Measles (morbillivirus)
Neisseria gonorrheoeae
Neisseria meningitidis
Plasmodium falciparum
Pseudomonas aeruginosa
Rhinovirus

Salmonella typhimurium
SARS-CoV-2
Staphylococcus aureus
Streptococcus pneumonia
Streptococcus pyogenes
Treponema pallidum
Trichophyton mentagrophytes
Varicella-Zoster virus

		FREE		

BEFORE WE START THE ACTIVITIES

Pre Assessment –

1) How many disease-causing agents (or their diseases) do you think can you name?
    a. None
    b. One or two
    c. Four or five
    d. Seven or eight
    e. More than a dozen

2) How many good or interesting roles for microbes do you think you can name?
    a. None
    b. One or two
    c. Four or five
    d. Seven or eight
    e. More than a dozen

3) How would you describe what a scientist looks like?

BEFORE WE START THE ACTIVITIES

Pre Assessment –

1) How many disease-causing agents (or their diseases) do you think can you name?
    a. None
    b. One or two
    c. Four or five
    d. Seven or eight
    e. More than a dozen

2) How many good or interesting roles for microbes do you think you can name?
    a. None
    b. One or two
    c. Four or five
    d. Seven or eight
    e. More than a dozen

3) How would you describe what a scientist looks like?

AFTER THE ACTIVITIES

POST Assessment –

1. How many disease-causing agents (or their diseases) do you think can you name?
    a. None
    b. One or two
    c. Four or five
    d. Seven or eight
    e. More than a dozen

2. How many good or interesting roles for microbes do you think you can name?
    a. None
    b. One or two
    c. Four or five
    d. Seven or eight
    e. More than a dozen

3. How would you describe what a scientist looks like?

AFTER THE ACTIVITIES

POST Assessment –

1) How many disease-causing agents (or their diseases) do you think can you name?
    a. None
    b. One or two
    c. Four or five
    d. Seven or eight
    e. More than a dozen

2) How many good or interesting roles for microbes do you think you can name?
    a. None
    b. One or two
    c. Four or five
    d. Seven or eight
    e. More than a dozen

3) How would you describe what a scientist looks like?
