# Supplementary material for: Shifting Perceptions about Microbes and Scientists: Reflections on Activities with High School Students
Source: Integr Org Biol. 2026 Mar 26;8(1):obag011. doi: 10.1093/iob/obag011 (PMC13048273; doi:10.1093/iob/obag011)
Supplement: obag011_Supplemental_Files [file obag011_supplemental_files.zip › Outreach activity scavenger hunt to go with poster set.pdf]

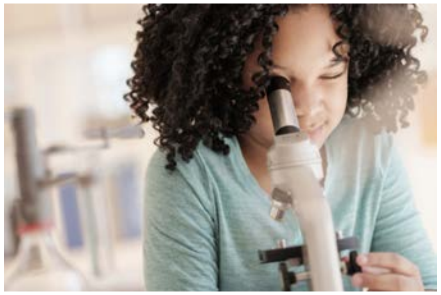
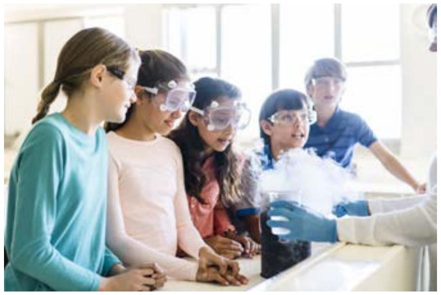
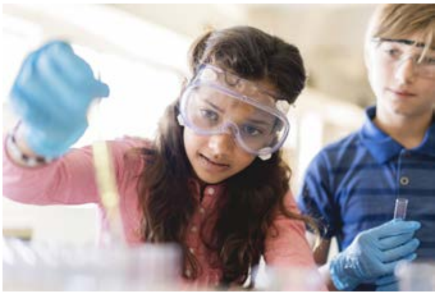
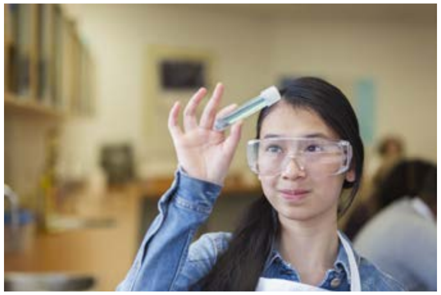

Picture Yourself as a Scientist!

- Science benefits when lots of different individuals are involved because people from different backgrounds think about problems in different ways and can come up with different approaches and solutions.

- Do you picture yourself as a scientist? What are your interests? What is your background?

- The **scientist scavenger hunt** activity introduces you to 12 contemporary scientists that you may not know. They come from various backgrounds, have different identities, and work in a range of science fields. After reading about them, fill in the missing word and decode the phrase.*

- Are you ready to picture yourself as a scientist?

*This activity was inspired by the scavenger hunts associated with the If/THEN #IfThenSheCan exhibit of women scientist 3D statues that was previously displayed at the Dallas Arboretum and Botanical Gardens and Washington, DC. You can take a virtual tour at https://ifthenexhibit.org/exhibit_preview/ or scan the QR code on the right.

Scavenger Hunt - **Find the scientist biographies, fill in the blanks, and decode the puzzle below with numbered letters in circles.**

___ ___ ___ ___ ___ ___ ___   ___ ___ ___ ___ ___   ___ ___ ___ ___   ___ ___ ___ ___ ___   ___ ___ ___ !

6 1 9 12 11 1 12   11 12 12 10 6   3 2 4 5   9 10 12 7 6   8 2 2

1. Aletha Maybank is a pediatrician and was on ___ ___ (___ 1) ___ ___ ___ ___ ___ ___ ___ ___ ___ ___.
2. Wendy Freedman uses ___ ___ ___ ___ ___ ___ (___ 2) ___ ___ ___ to measure the size of the universe.
3. André Isaacs is a ___ ___ ___ ___ ___ ___ ___ ___ (___ 3) professor who wears bright lab coats & dances.
4. Charles Limb is a hearing specialist who studies people's brains when they make up ___ (___ 4) ___ ___ ___.
5. Sara Rankin is looking for new ___ (___ 5) ___ ___ ___ that help people get better after injuries.
6. Donna Shaver studies and saves ___ ___ ___ ___ ___ ___ (___ 6) at national seashore parks.
7. Gemma Reguera uses ___ (___ 7) ___ ___ ___ ___ ___ ___ to clean up nuclear waste sites.
8. Hayat Sindi is creating affordable tests for people to check their ___ ___ ___ ___ (___ 8) ___ from home.
9. Katalin Karikó studies of RNA (ribonucleic acid) molecules led to a COVID-19 ___ ___ ___ ___ (___ 9) ___ ___ .
10. Lydia Villa-Komaroff showed how bacteria make insulin for people with (___ 10) ___ ___ ___ ___ ___ ___ ___.
11. Darlene Cavalier created groups to promote STEM women and citizen ___ ___ ___ ___ (___ 11) ___ ___.
12. Sam Long is a high school ___ (___ 12) ___ ___ ___ ___ ___ who helps students think about genetics.
