# Supplementary material for: Shifting Perceptions about Microbes and Scientists: Reflections on Activities with High School Students
Source: Integr Org Biol. 2026 Mar 26;8(1):obag011. doi: 10.1093/iob/obag011 (PMC13048273; doi:10.1093/iob/obag011)
Supplement: obag011_Supplemental_Files [file obag011_supplemental_files.zip › Outreach activity slide show.pdf]

Introduction to Microbiology & Scientists

Outreach activity

2.5 hours

Activities plan

1. Quick pre-survey
2. Disease-causing fuzzy microbe information scavenger hunt (complete matching handout)
3. Review of disease agents (via Bingo activity)
4. Small group break out (do both in smaller groups)
   A. Out of room: Tour of hall scientist posters & scavenger hunt
   B. In room: Microbe video (https://www.youtube.com/watch?v=5DTrENdWvvM) & photo booth station (optional)
5. Slides, visuals, & group discussion for good microbes
6. Quick post-survey

Amazing Microbes (and the researchers who study them)

Inspired by the 2024 coloring book from the American Society for Microbiology

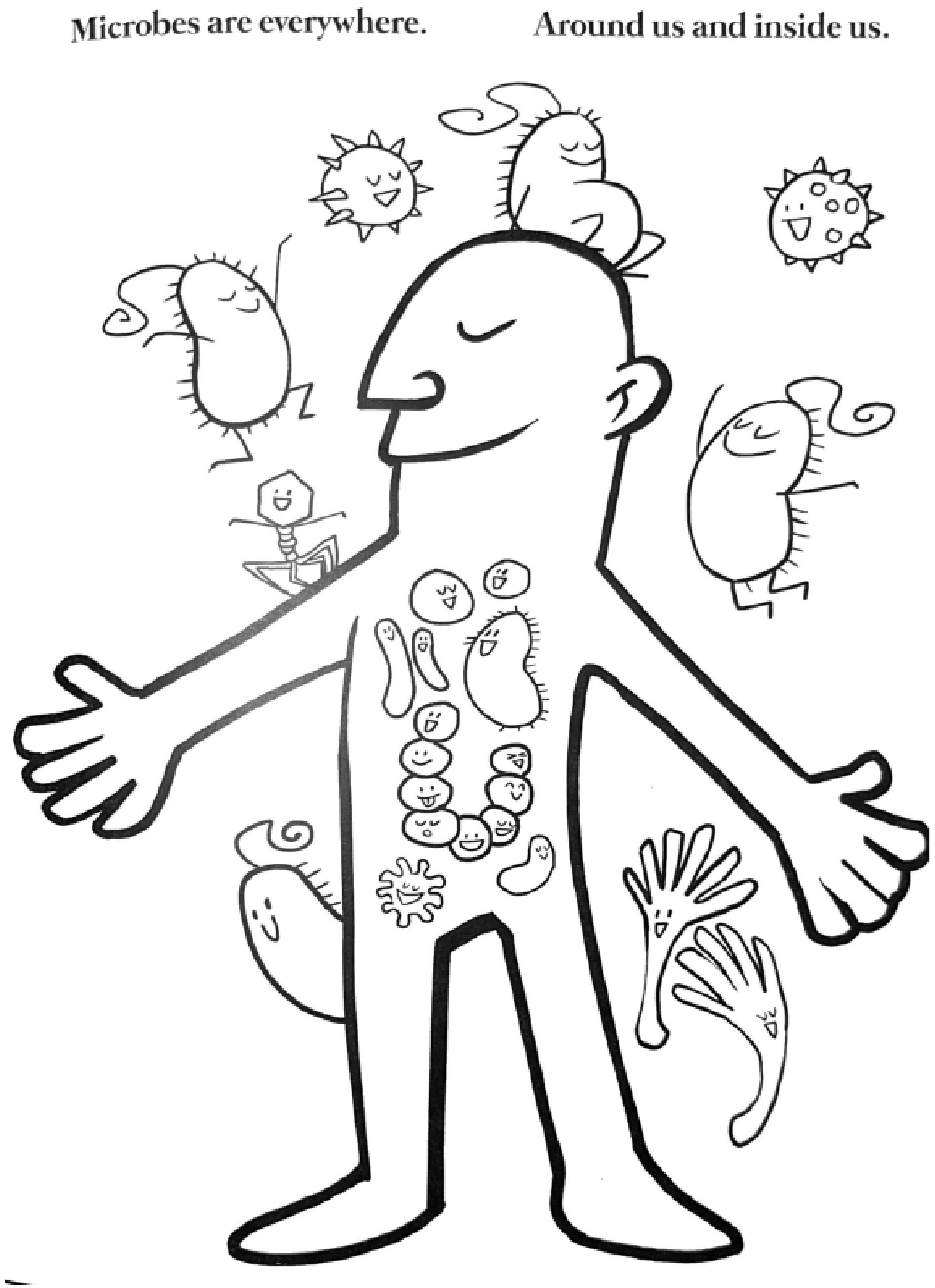

- As you saw in the video, microbial cells outnumber our body cells about 10 to 1.

- *Have you heard that before?*
- *It kind of makes you consider what makes you YOU?*

- *Do you know of other foods that make use of microbes in some way?*

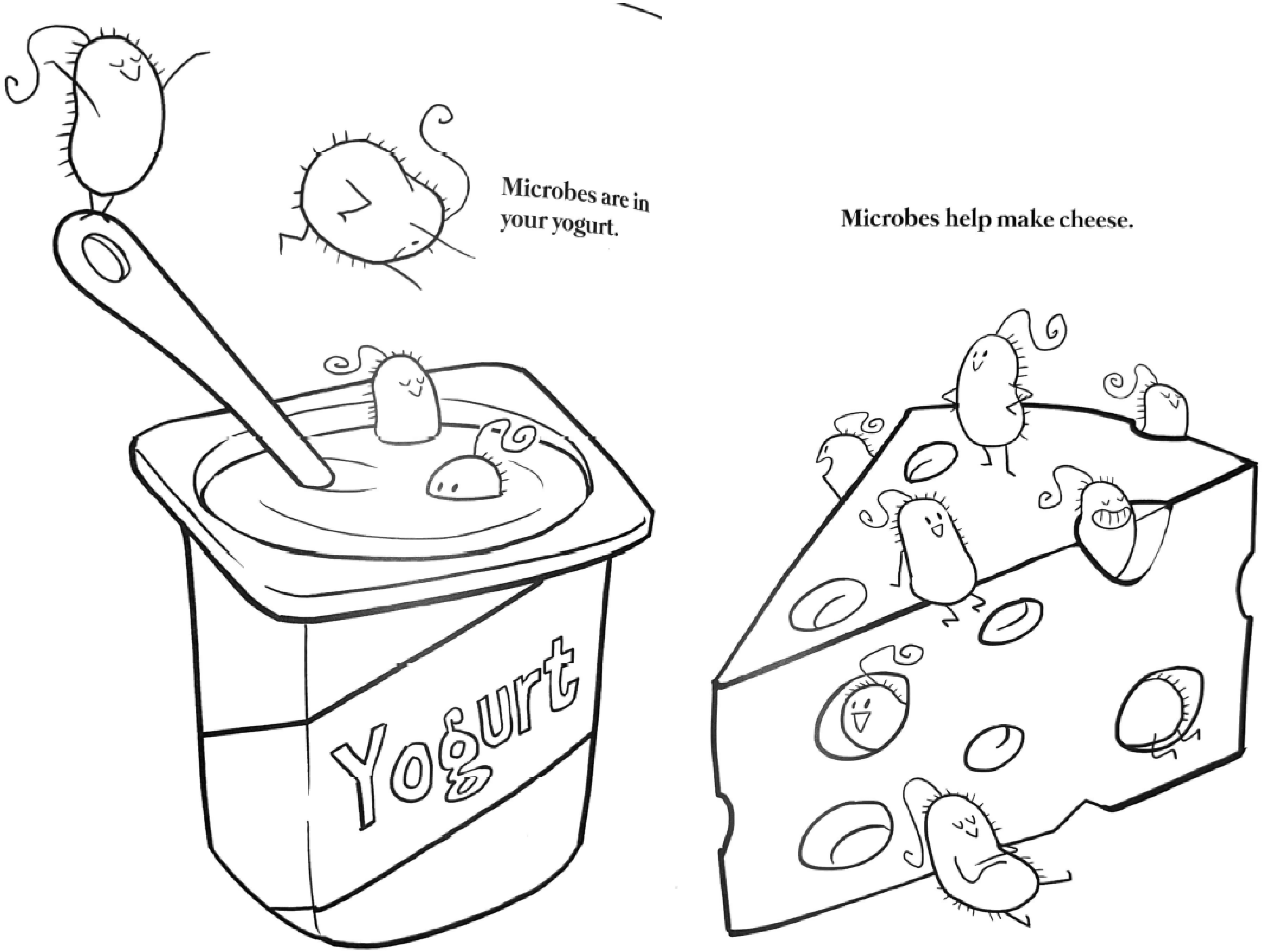

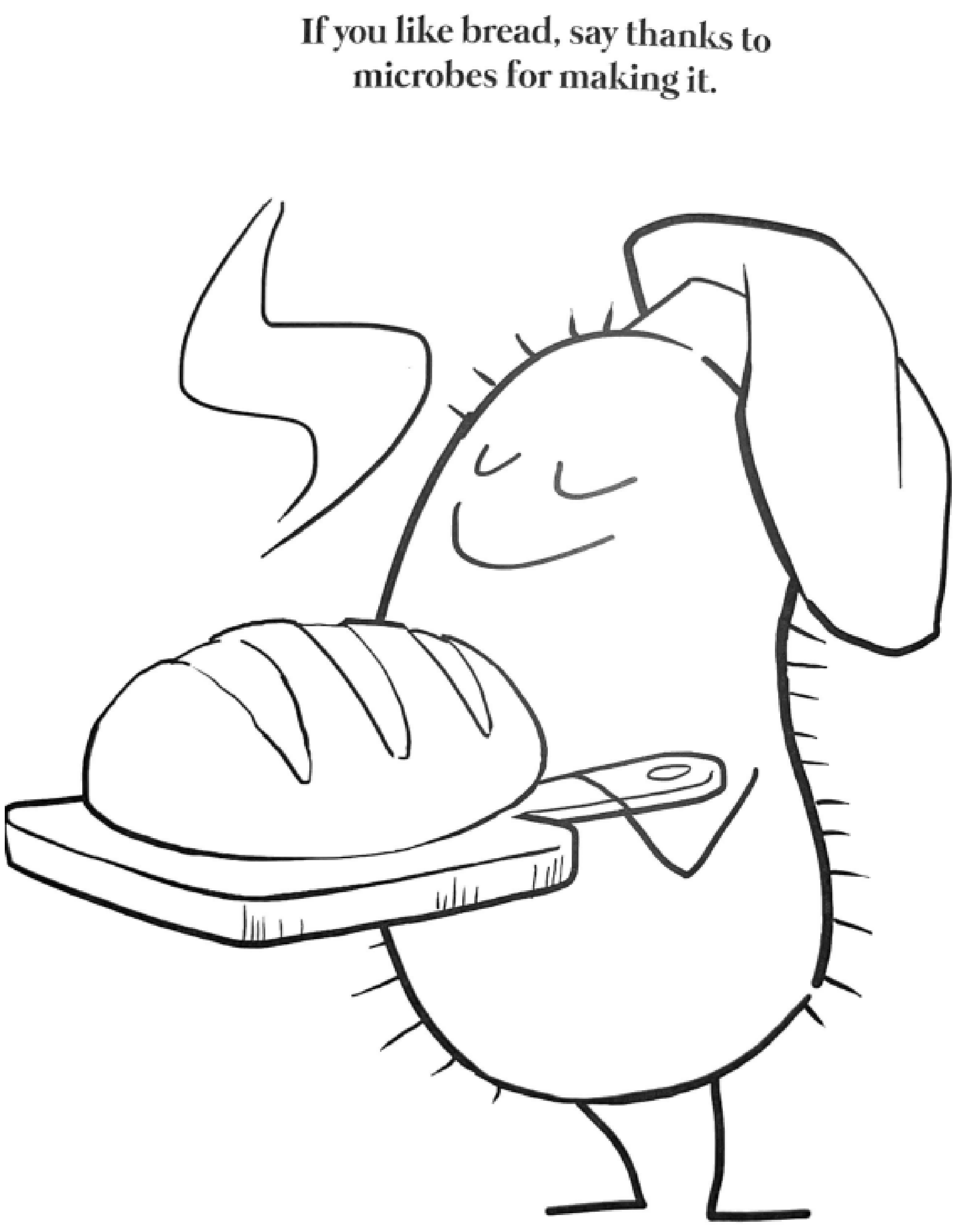

- *Saccharomyces cerevisiae* break down sugars to gas bubbles (that make bread rise) and alcohol (that is cooked off).

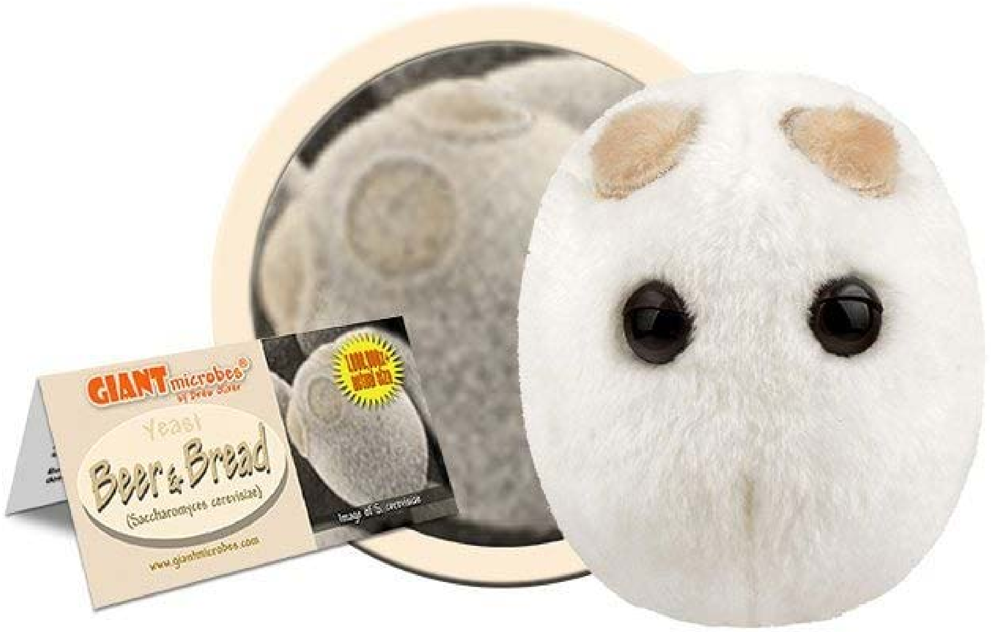

- *Escherichia coli* help digest food and make vitamins for us.

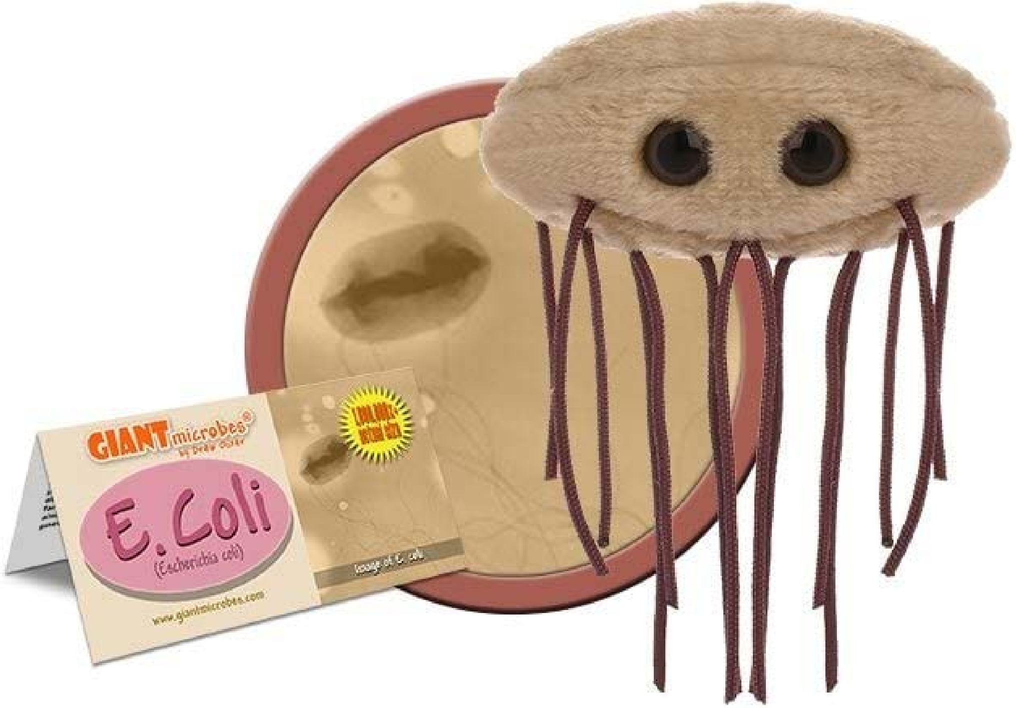

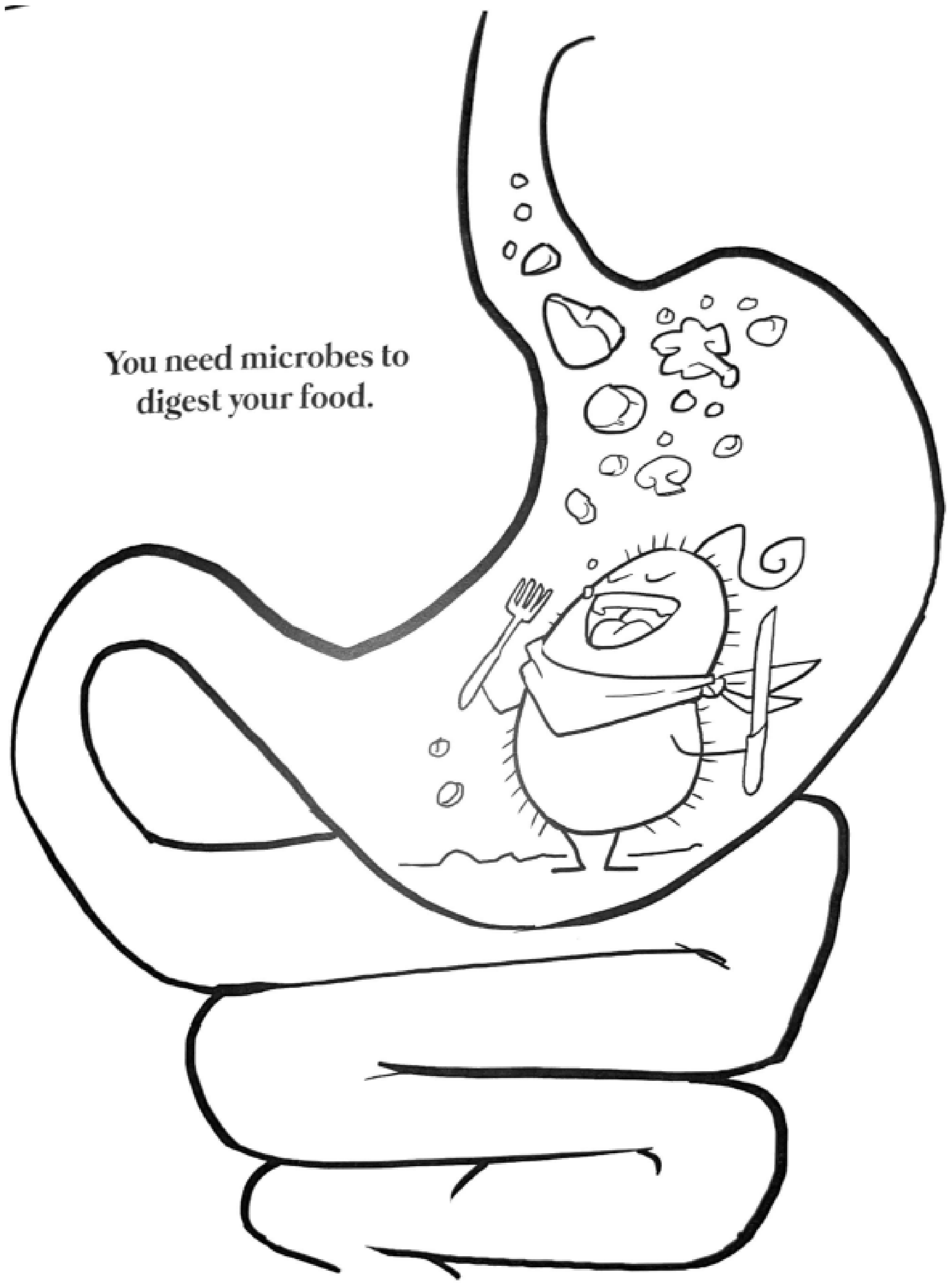

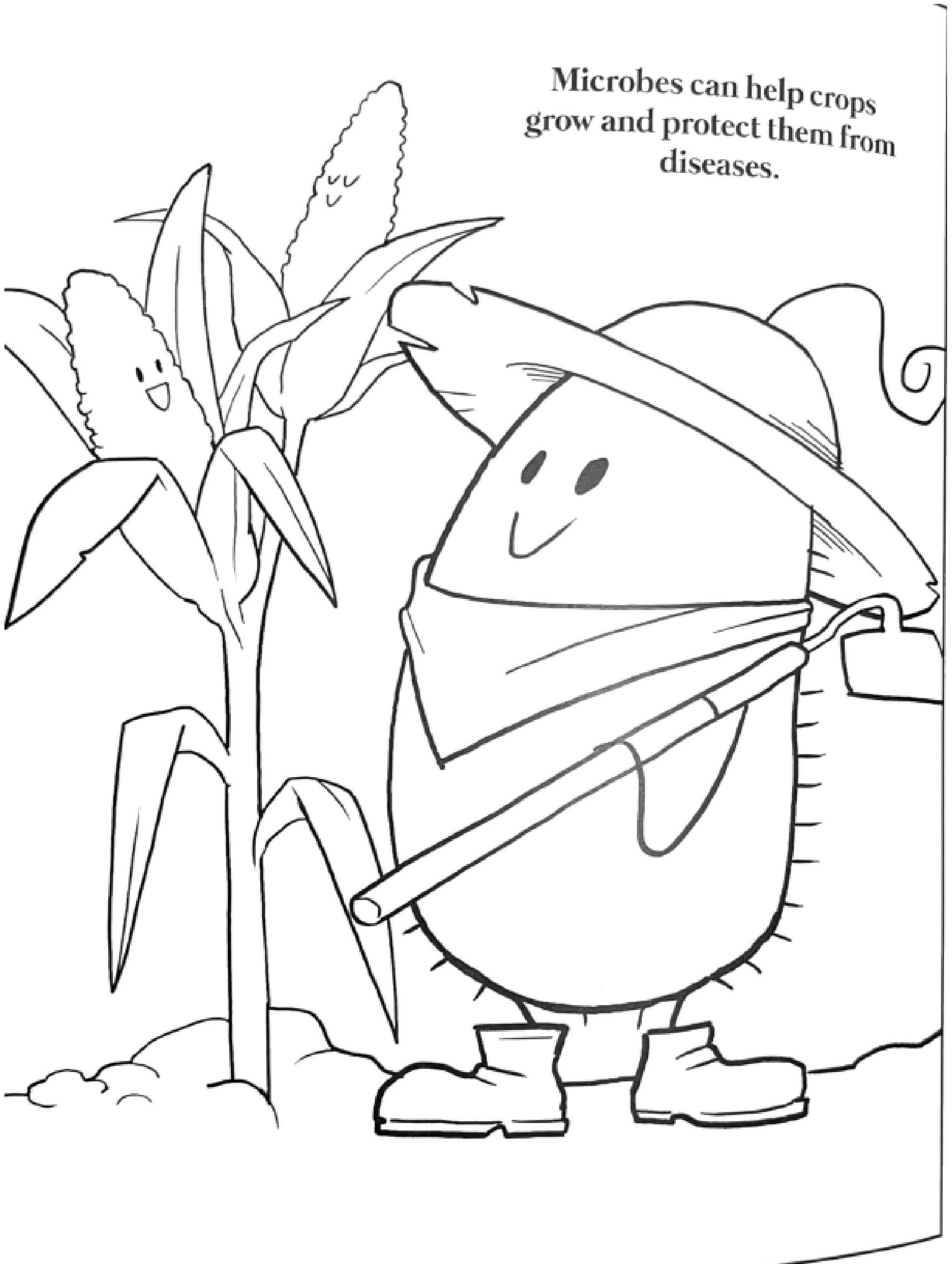

- *Anyone from farming families have any experience with this concept?*

- For example, some microbes make cellulase, which can break down cellulose in clothes in the form of grass stain etc.

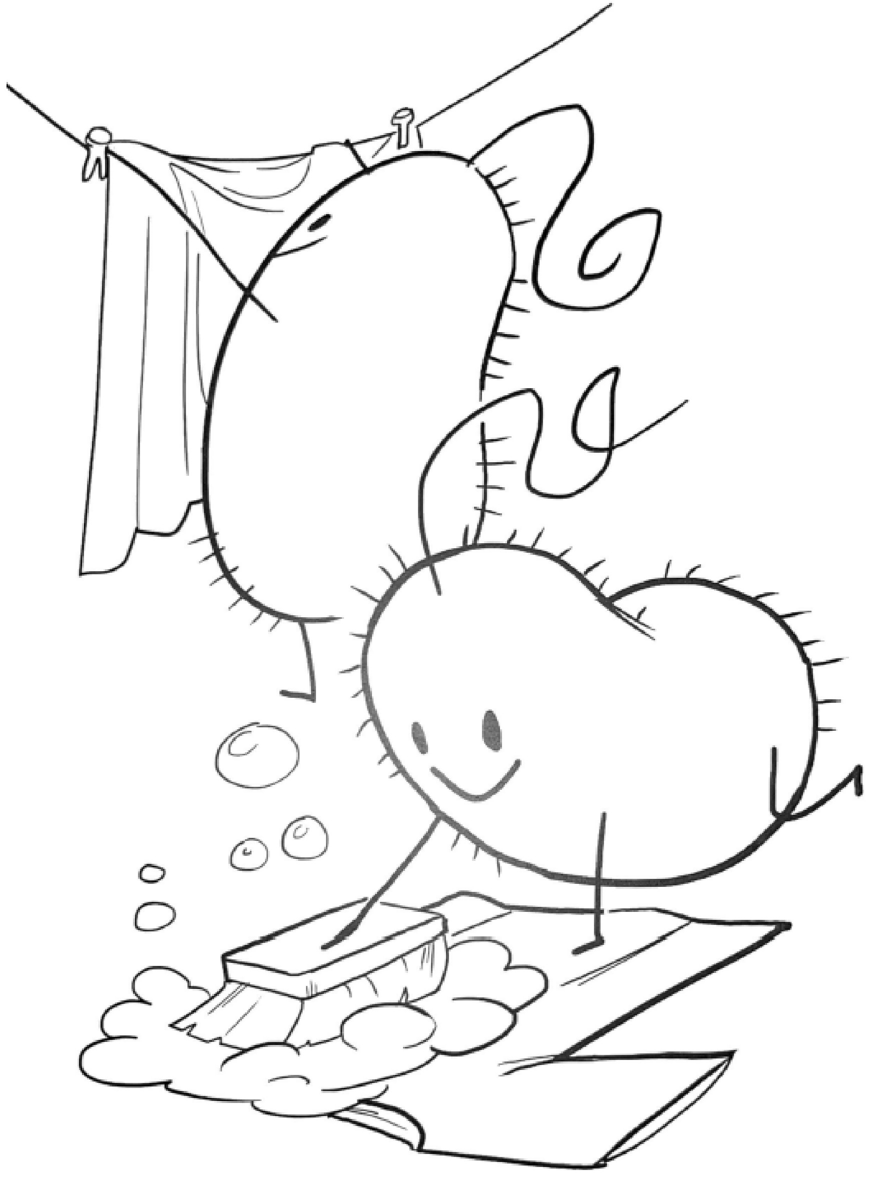

The laundry detergent that cleans your clothes contains products from microbes.

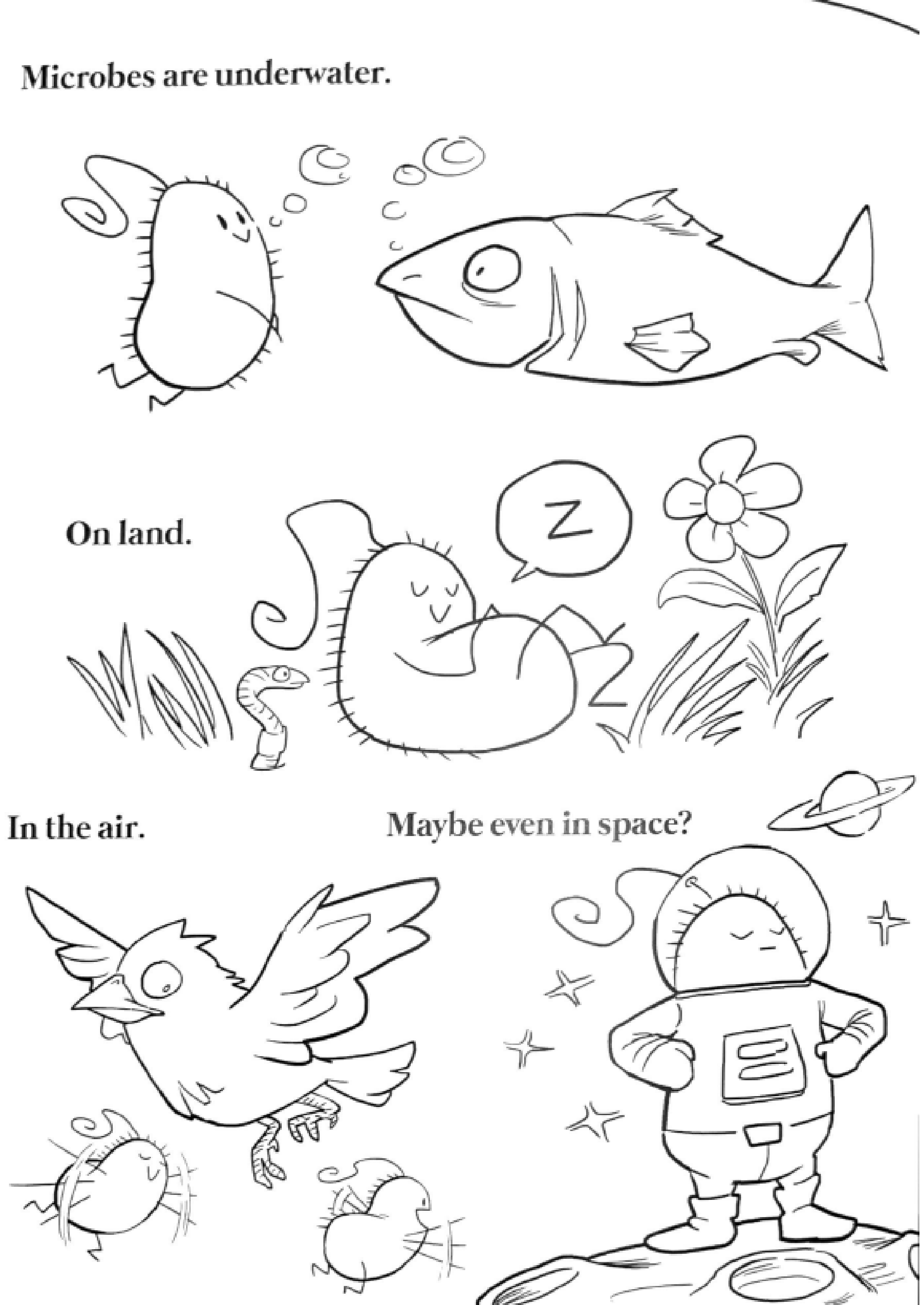

- This makes me think of bioremediation.
- *Does anyone want to guess about that term and some applications?*

- We can make use of some of the enzymes or products some of these high-temperature bacteria (and archaea).

- For example, we use DNA polymerase from *Thermus aquaticus* (*Taq*) in polymerase chain reactions (PCR) to help make more copies of DNA of interest to study all sorts of things in science.

Microbes can live in volcanoes.

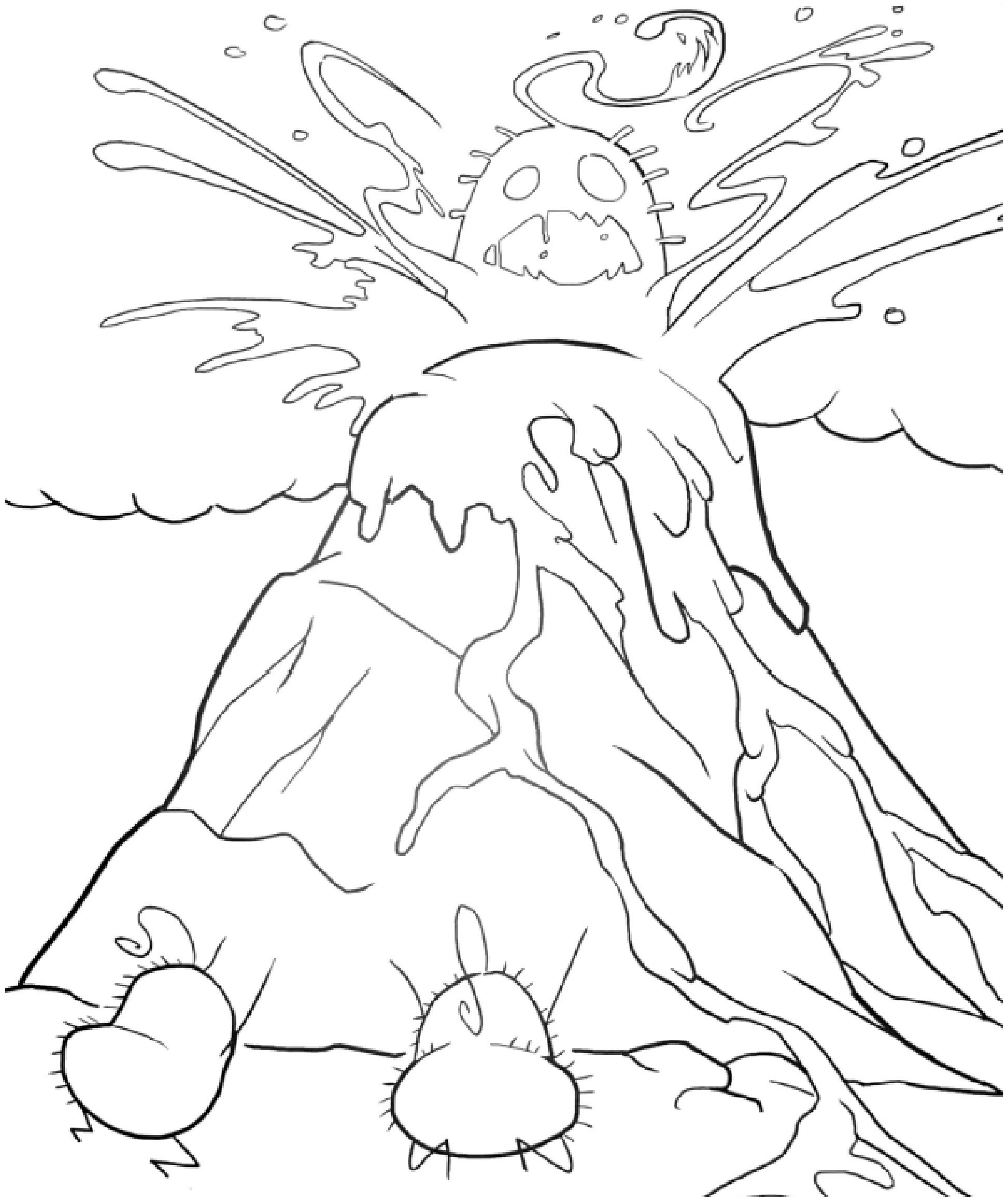

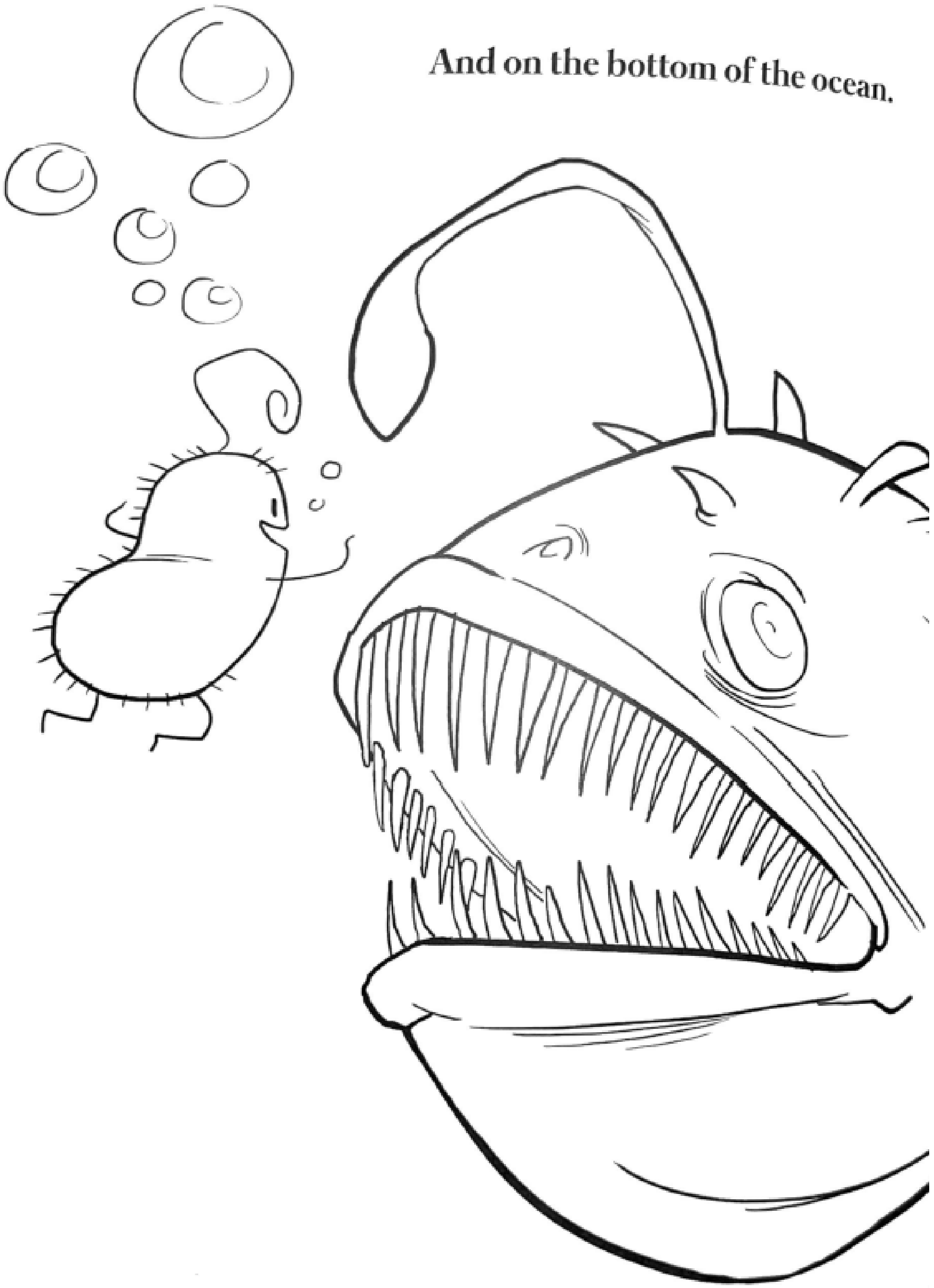

- These bacteria have to withstand high water pressure and often extreme temperatures.
- Diatoms are marine algae (a type of microbe not yet mentioned). Diatomaceous earth powder from fossilized diatoms is used to filter pool water and other applications.

Microbes started life on our planet.

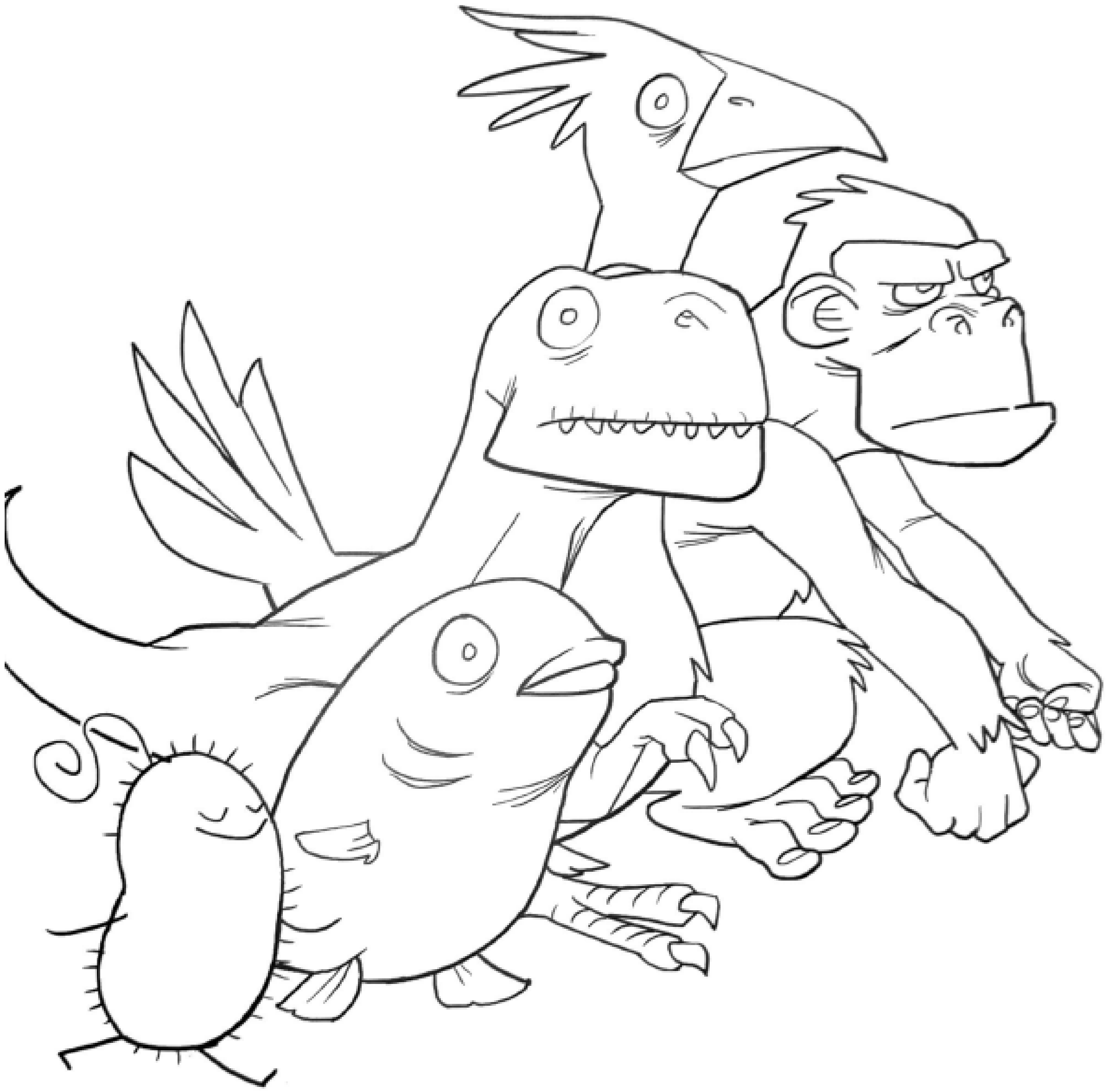

- This relates to the theory of endosymbiosis and the origins of eukaryotes from prokaryotes.

- *Has anyone heard that term?*
- *Any ideas what it may mean?*

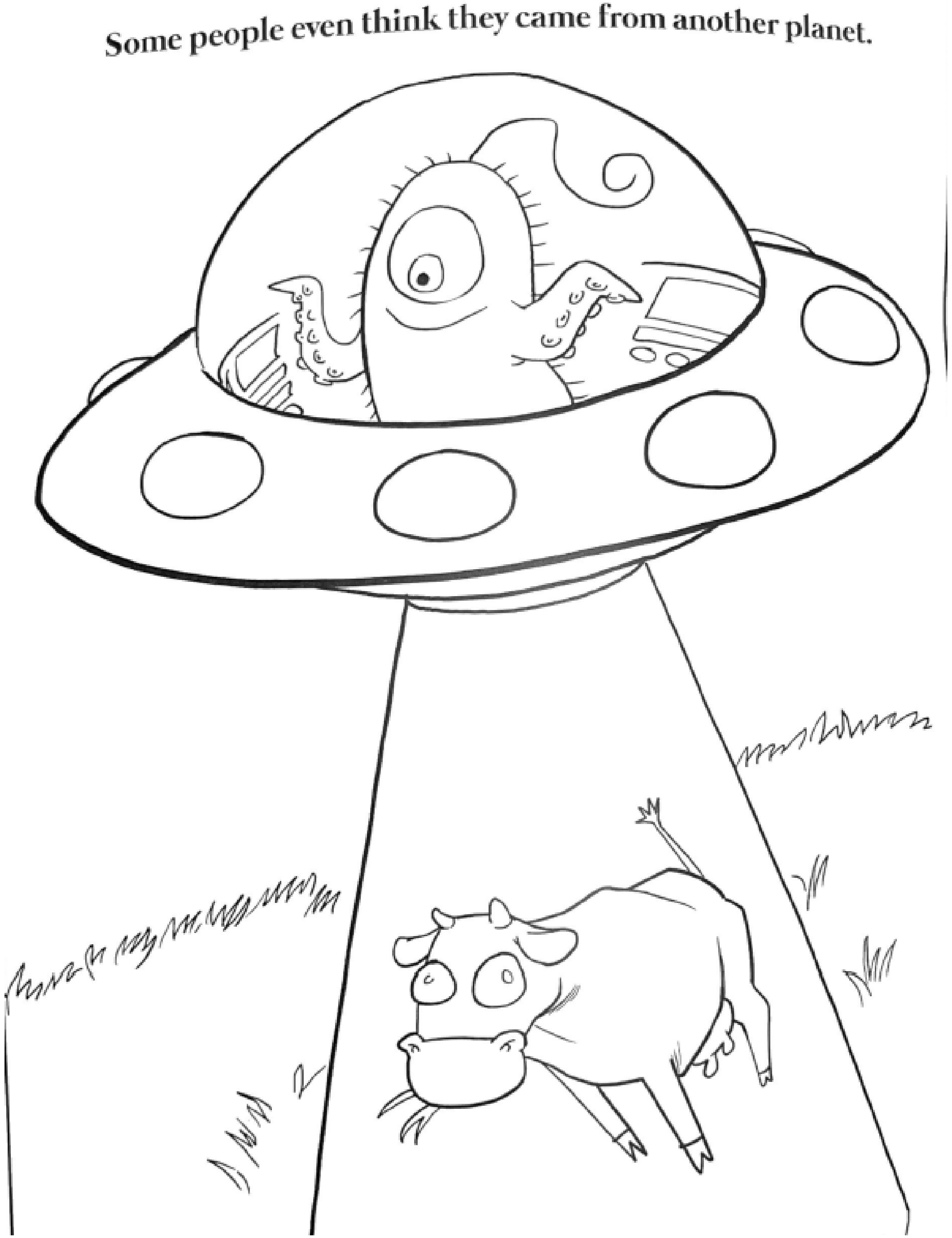

- *What do you think?*
- *What would this require?*

- Discuss personal research interests and others of possible interest to audience.

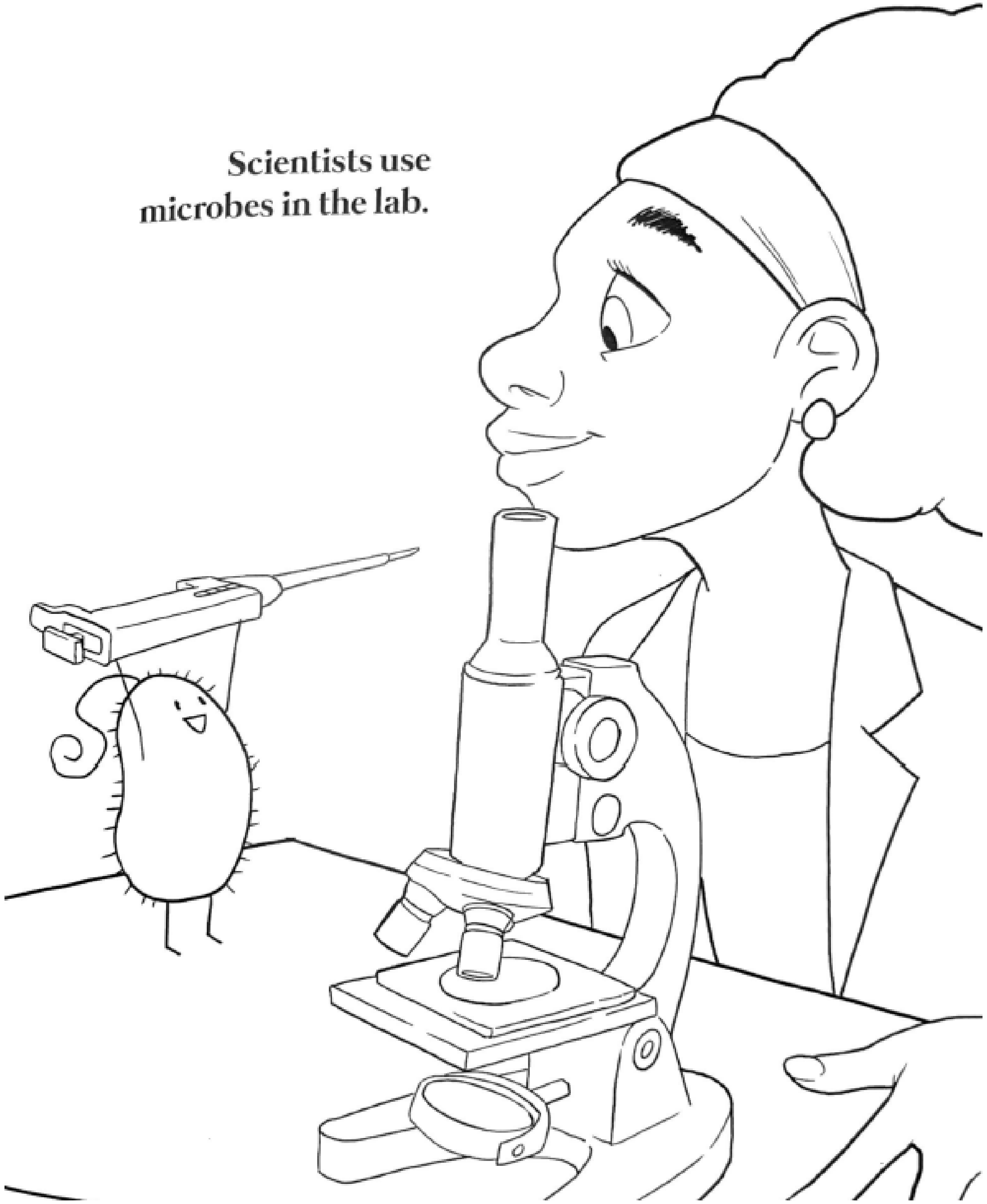

We can use microbes to make things like insulin to treat diabetes.

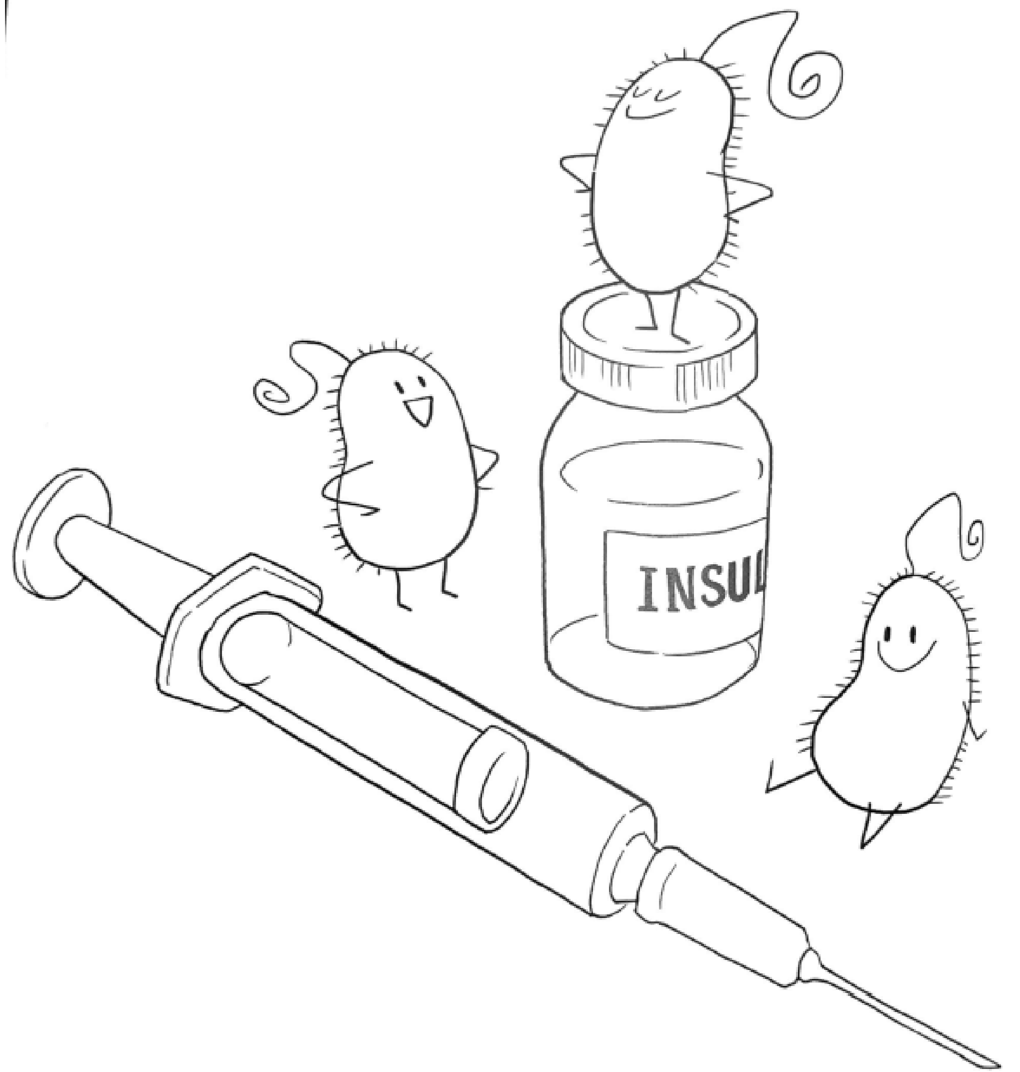

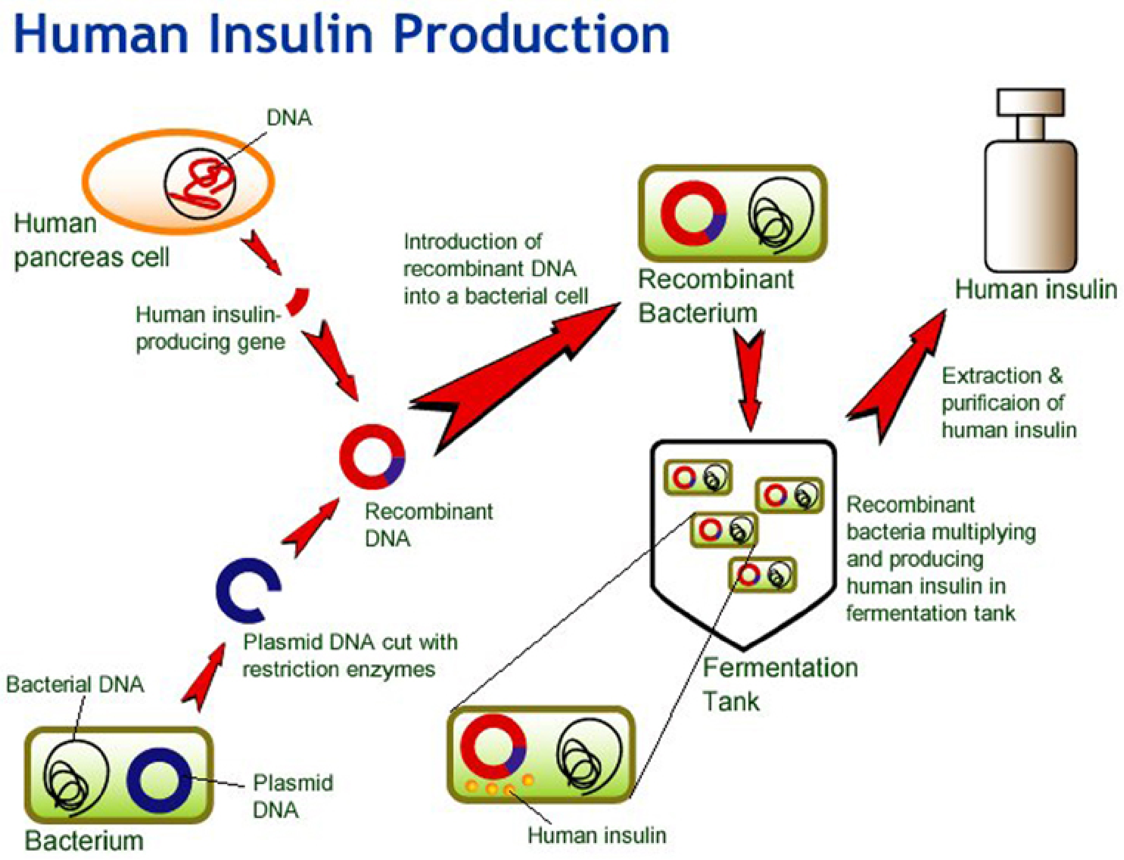

https://www.eduhk.hk/biotech/eng/classrm/class_health5.html

- Penicillin comes from a fungus, but many bacteria make antibiotics as well.

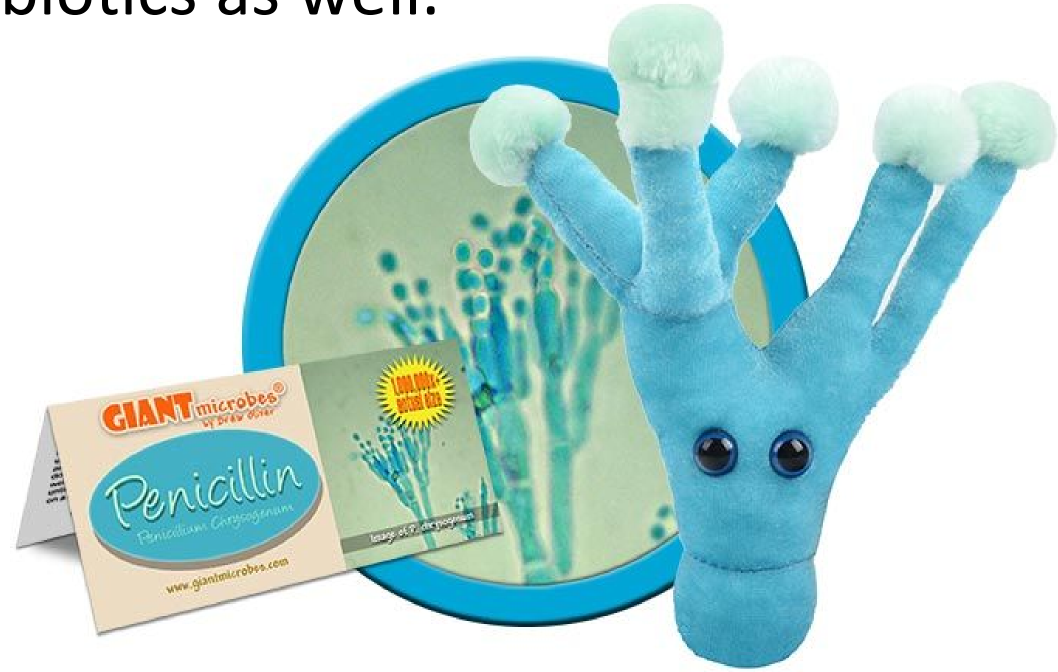

- *Why do you suppose bacteria in the soil might make antibiotics?*

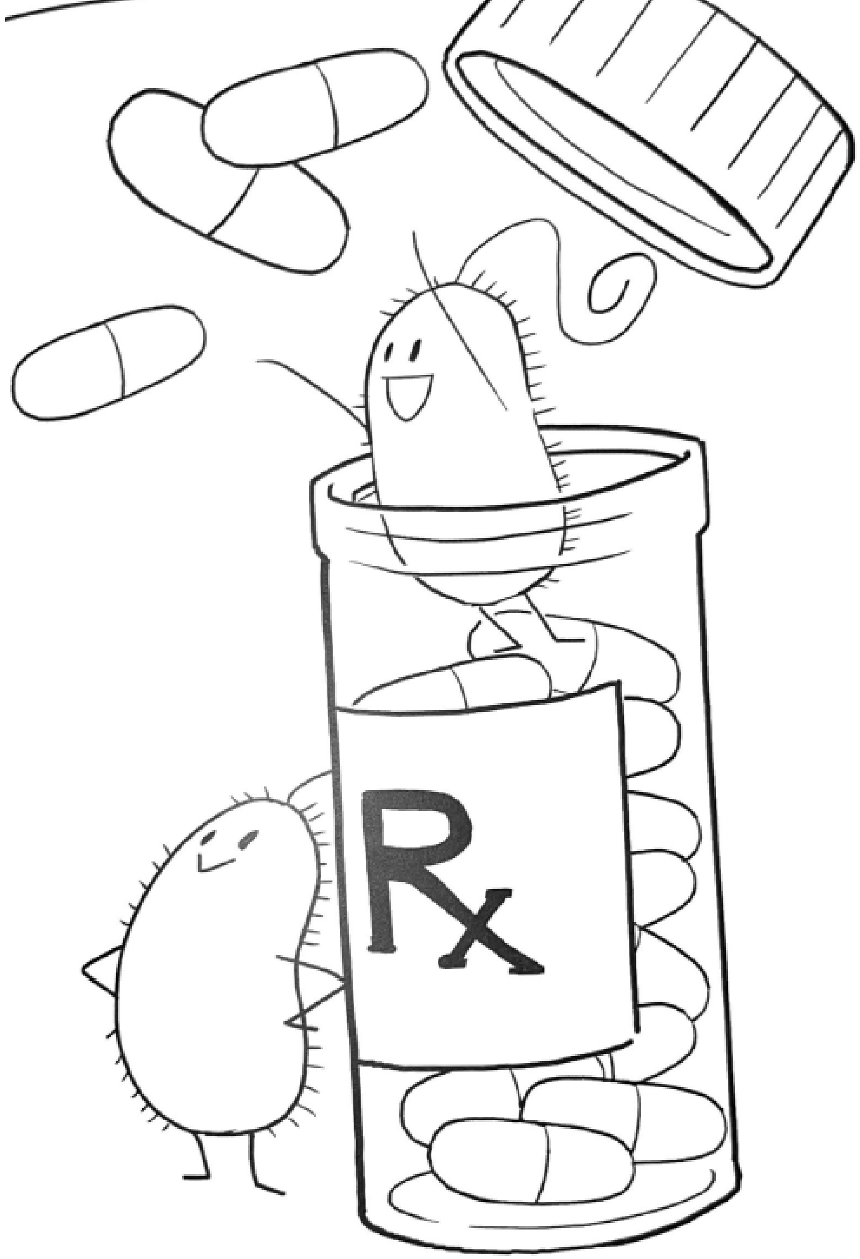

Microbes helped scientists discover the antibiotics you take when you get sick.

We can also use microbes to study how diseases like COVID-19 spread.

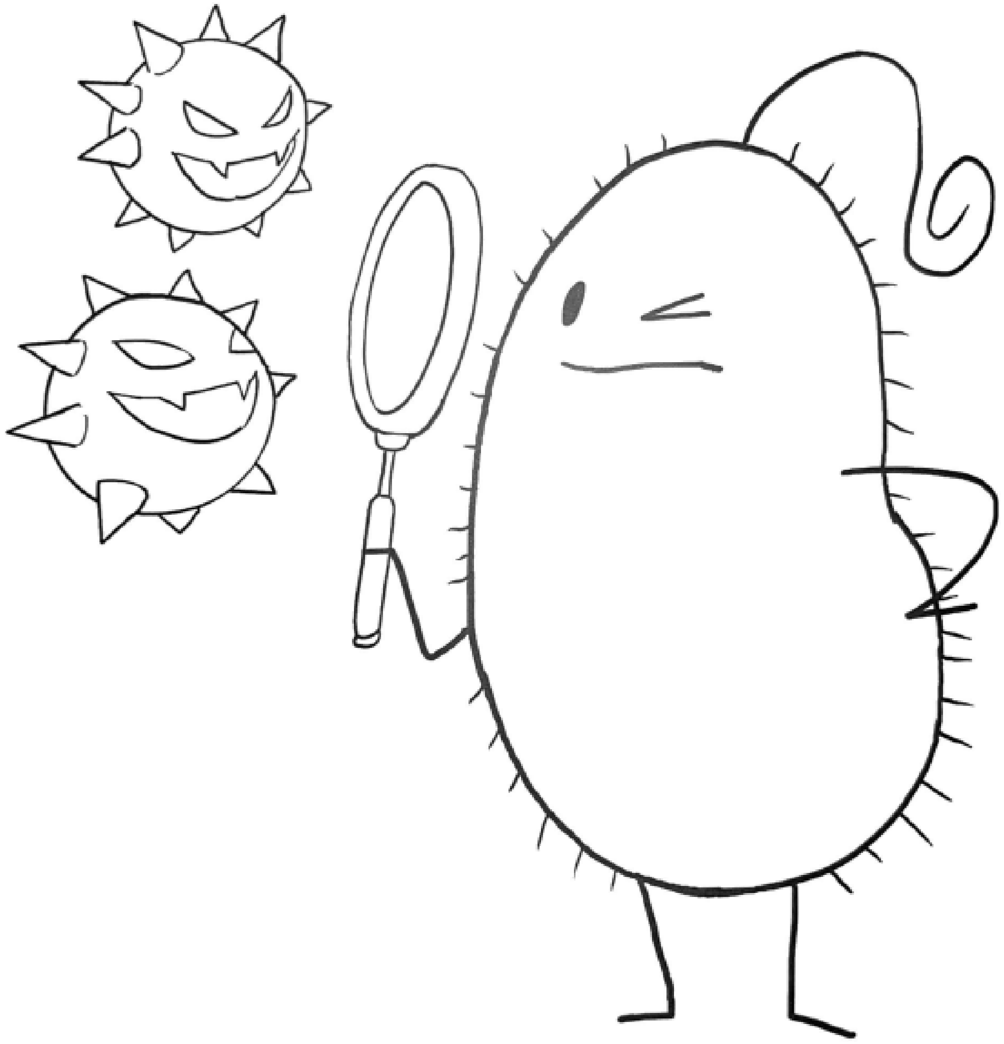

- It is fortunate that different people were already studying both coronaviruses and RNA methods that eventually lead to COVID-19 vaccines!

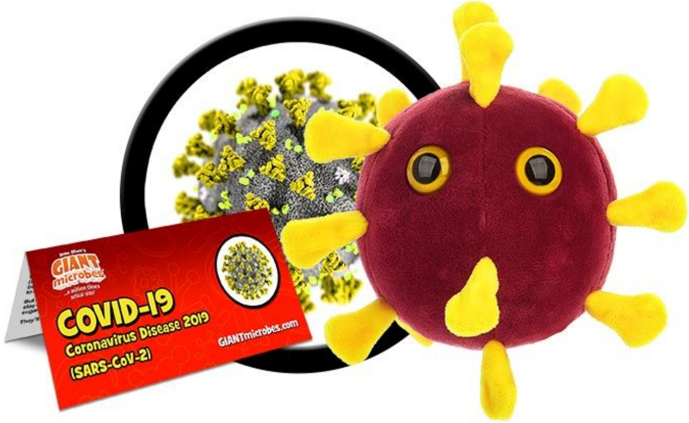

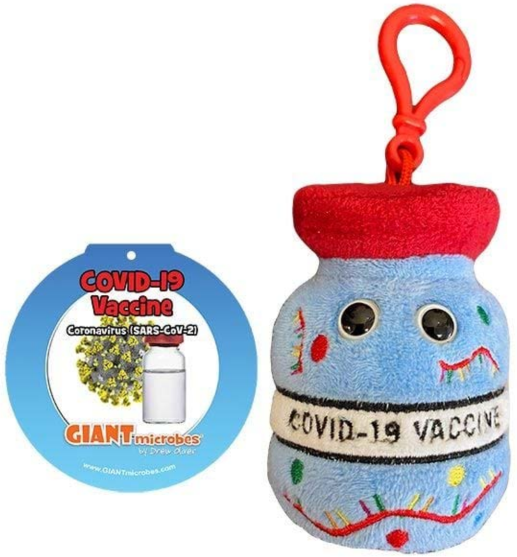

Some people even use microbes to make art.

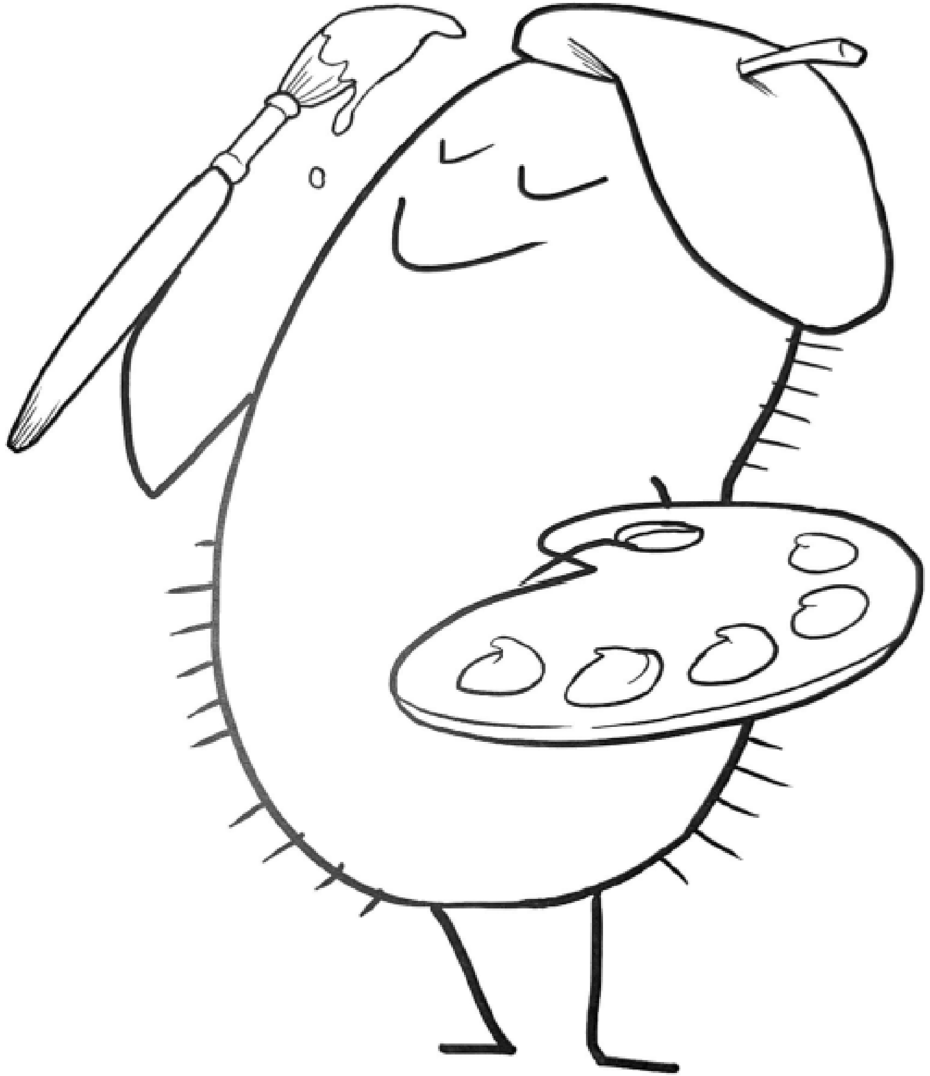

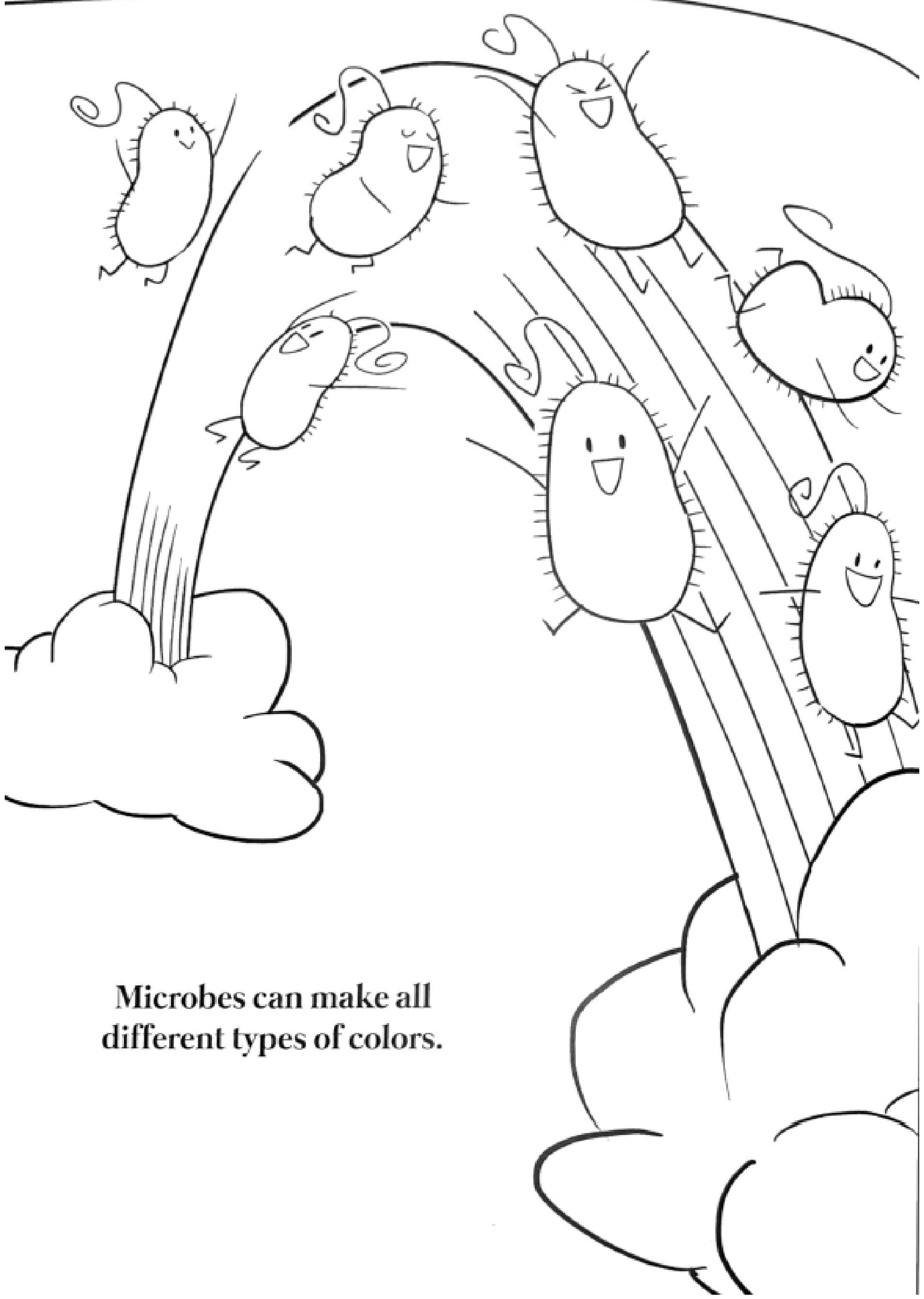

Microbes can make all
different types of colors.

What Is 'Agar Art'?

Have you ever seen art created in a petri dish using living, growing microorganisms? That's agar art! Creators use either naturally colorful microbes, like the red bacteria *Serratia marcescens*, or genetically modified microbes, like the yeast *Saccharomyces cerevisiae* transformed with violacein genes, as 'paint' and various types, shapes and sizes of agar as a 'canvas.' In fact, the original agar artist was none other than Alexander Fleming himself!

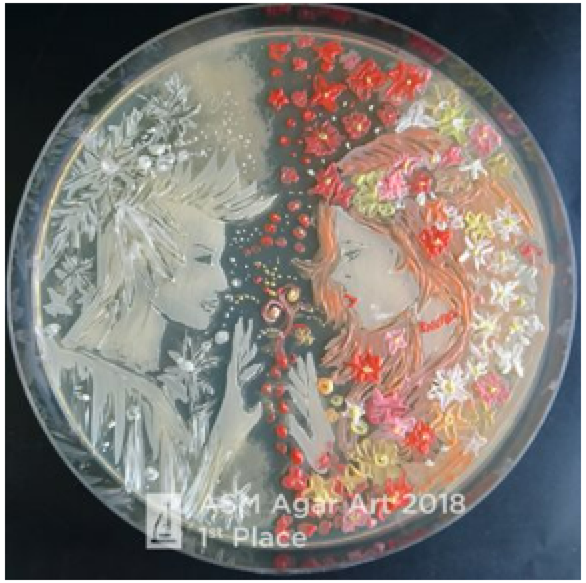

ASM Agar Art Contest 2018, 1st Place. "The battle of winter and spring," Ana Tsitsishvili, Undergraduate Student, Agricultural University of Georgia, Tbilisi, Georgia.

https://asm.org/events/asm-agar-art-contest/home

Alien Medley

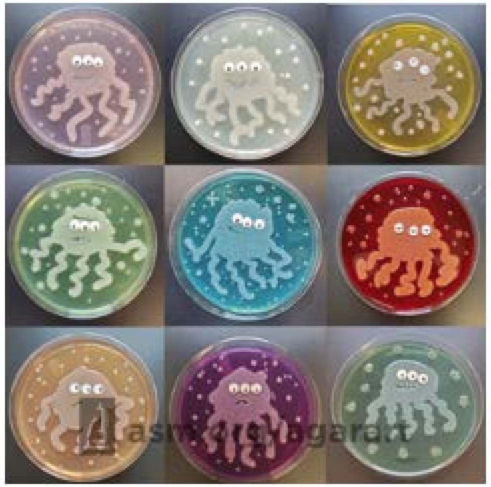

Artist: Ella Rotman (University of Chicago)
Source: American Society for Microbiolog
y

Agarnaut

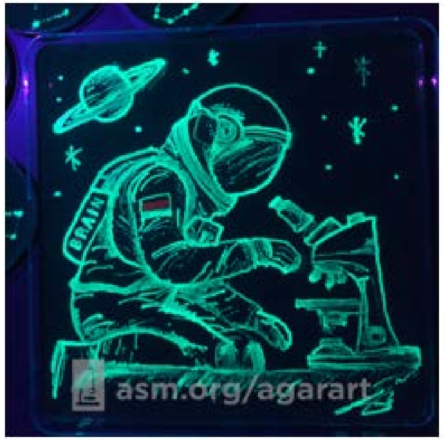

Artist: Svenja Ries (BRAIN Biotech AG)
Source: American Society for Microbiolog
y

Microbial Perseverance

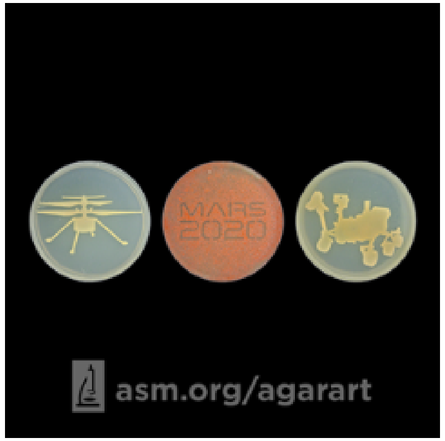

Artists: Amelia Cox-Fermandois, A. Victoria Suárez-Clerc, Hazajem Colque-Ferrer, Camilo Berríos-Pastén and Andrés E. Marcoleta
Source: American Society for Microbiolog
y

A Cosmic Journey of Yeast Cells.

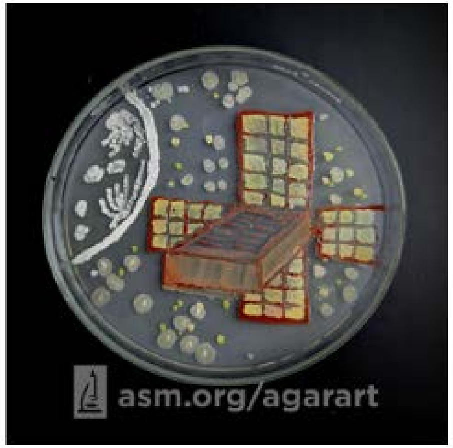

Artist: Shashank Yadav (Amity University Mumbai, India)
Source: American Society for Microbiolog
y

Shapeship Flying Through the Stars

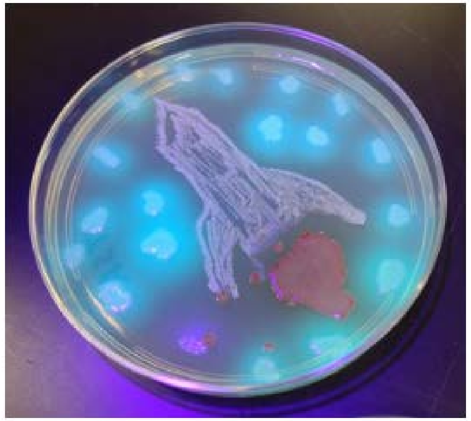

Artist: Rachel Moore (Angelina College)
Source: American Society for Microbiology

The Birth of a Star

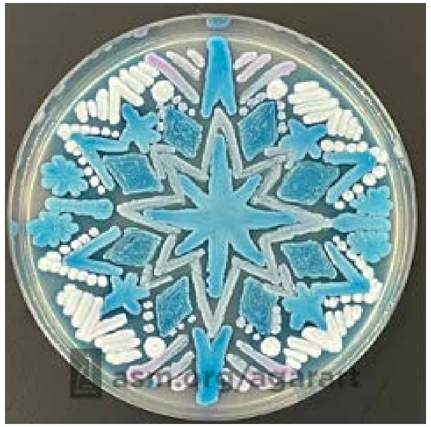

Artist: Hina Uchikawa (Fukushima Medical University)
Source: American Society for Microbiolog
y

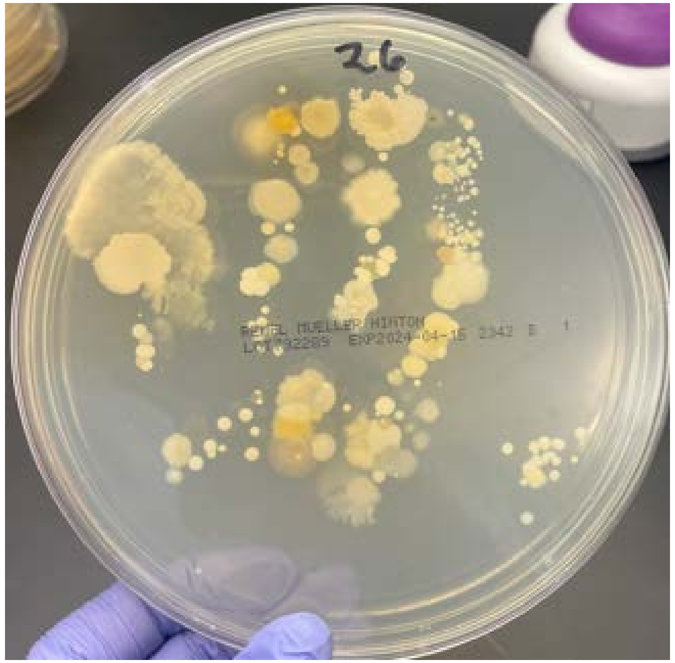

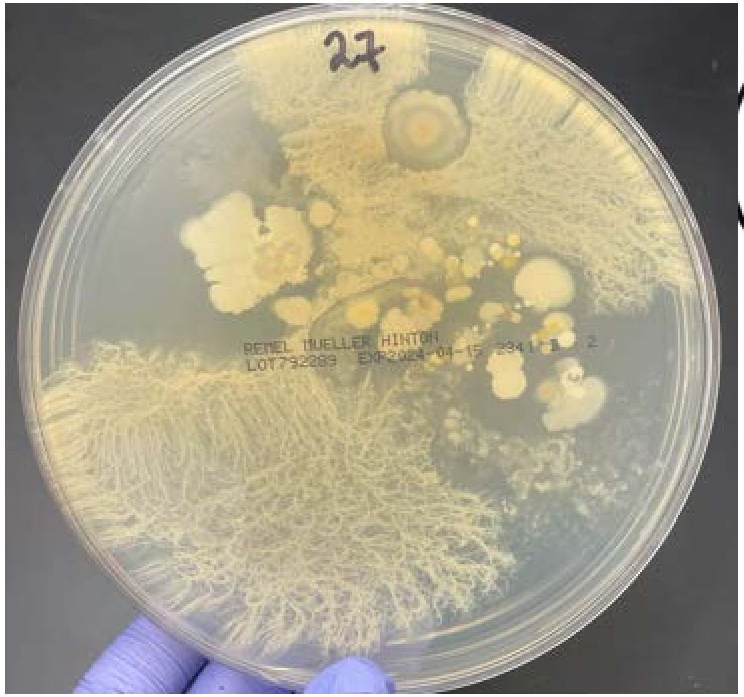

They can grow in
different patterns
and shapes.

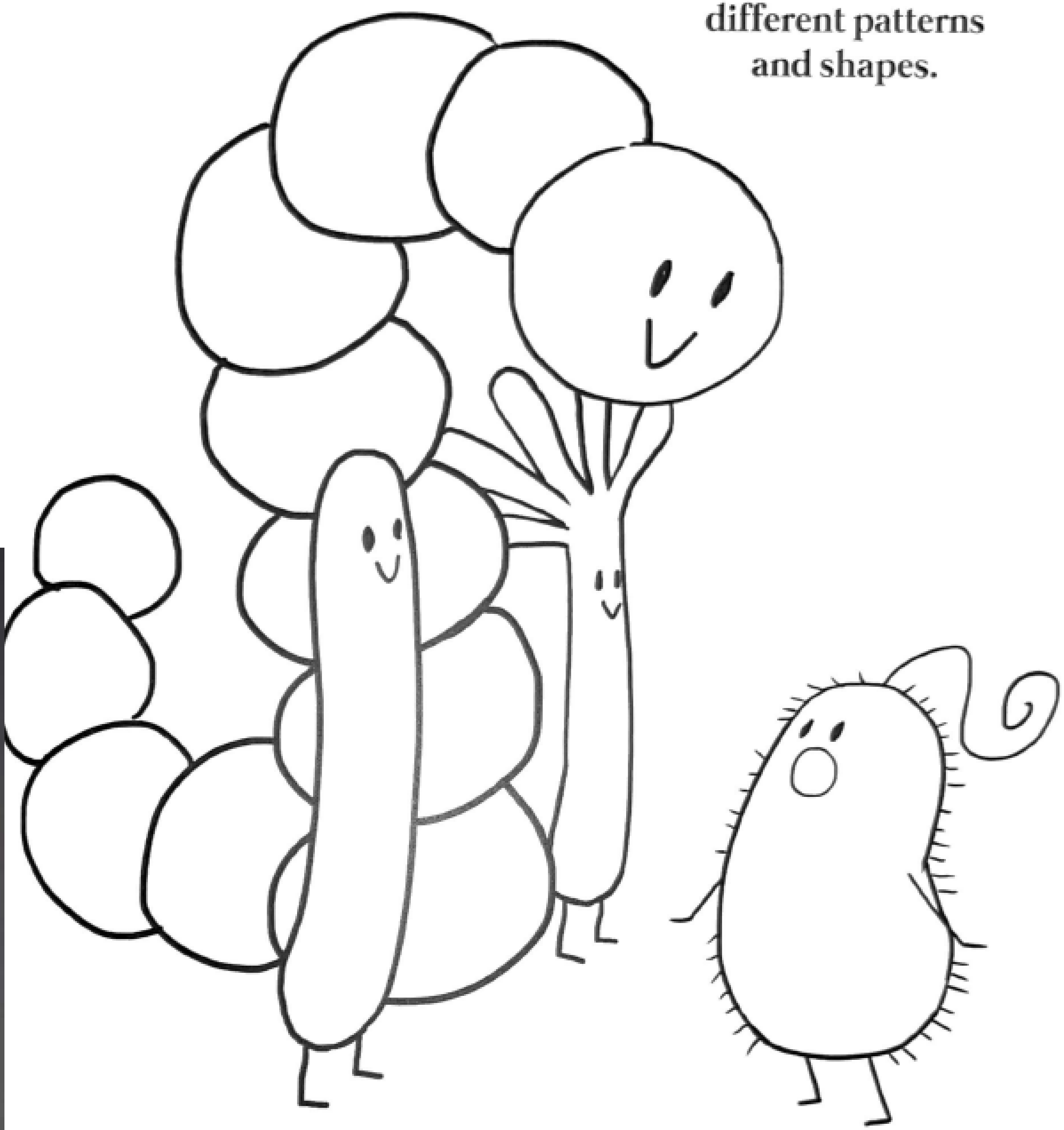

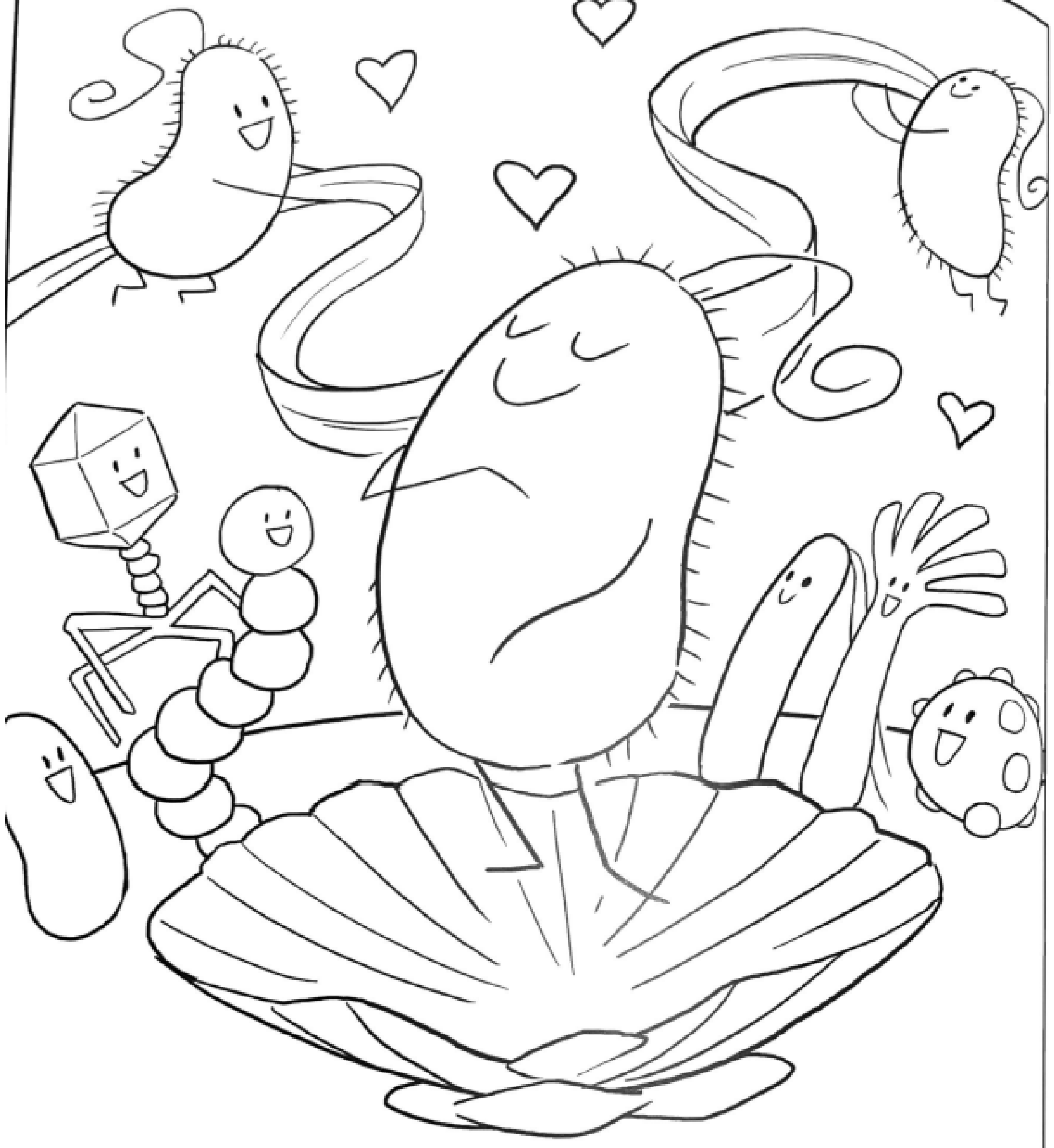

For all of these reasons, we think microbes are beautiful.

- *What do you think about microbes now?*
