# Supplementary material for: Shifting Perceptions about Microbes and Scientists: Reflections on Activities with High School Students
Source: Integr Org Biol. 2026 Mar 26;8(1):obag011. doi: 10.1093/iob/obag011 (PMC13048273; doi:10.1093/iob/obag011)
Supplement: obag011_Supplemental_Files [file obag011_supplemental_files.zip › Outreach- scientist poster example - with scientist permission.pdf]

Dr. Lydia Villa-Komaroff, Ph.D.

Molecular Biologist (studies & edits DNA)

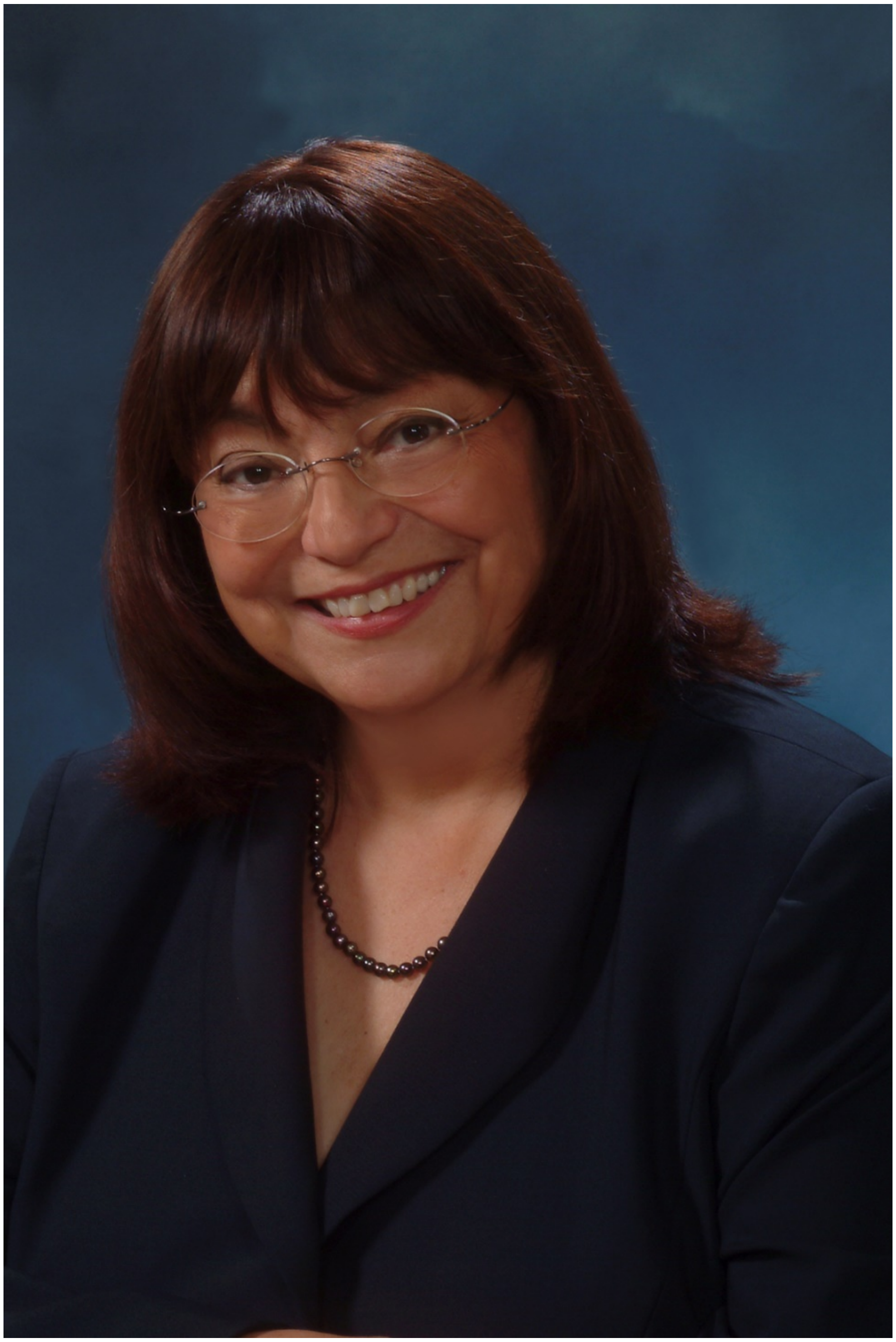

Figure 1 provided by Lydia Villa-Komaroff

About Dr. Villa-Komaroff and how she got into science:

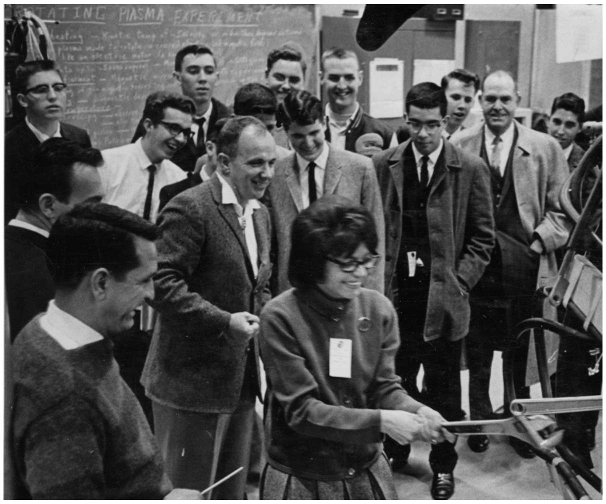

Figure 2 provided by Lydia Villa-Komaroff from a high school trip to Los Alamos, she is in the front

- She wanted to be a scientist since she was 9 and grew up in Santa Fe, NM.
- When she went to school, girls were not always encouraged to pursue science and she was often the only girl in the class (Figure 2). She found the biology classes exciting and welcoming, so that is how she picked her major in college.
- In 1975 she was the one of the first Mexican American woman to earn a Ph.D. in science in the United States.

What is her job and what does she do?

- She now runs her own company called Intersections SBD Consulting that brings together her science (S) experiences from running a laboratory, business (B), and advocating for diversity (D) in science.
- She was the lead author of the paper that showed how bacteria could be used to make insulin for people with diabetes who needed it by putting the human DNA into the bacteria and turning the bacteria into insulin factories.
- She is a co-founder of The Society for the Advancement of Chicanos/Hispanics and Native Americans in Science (SACNAS).

Want to know more about her?

- Scan the left QR to see her featured in *"The Wonder Woman Project"* series.
- Explore her Wikipedia page at https://en.wikipedia.org/wiki/Lydia_Villa-Komaroff
